# Supplementary material for: Use of social media in recruiting young people to mental health research: a scoping review
Source: BMJ Open. 2023 Nov 28;13(11):e075290. doi: 10.1136/bmjopen-2023-075290 (PMC10685975; doi:10.1136/bmjopen-2023-075290)
Supplement: Supplementary data [file bmjopen-2023-075290supp001.pdf]

Supplementary Materials

Supplementary Material 1: Full electronic search strategy by database

Database: PUBMED
Limitations:

- 2011 – Present
- Humans
- English
- Adolescent 13-18

Search Terms:
((((((((((((((((adolescent[Title/Abstract]) OR (adolescent[MeSH Major Topic])) OR (youth[Title/Abstract]) OR (teenager[Title/Abstract])) OR ("young adult"[Title/Abstract])) OR (young adult[MeSH Major Topic])) AND ("mental health"[Title/Abstract])) OR (mental health[MeSH Major Topic])) OR ("mental illness"[Title/Abstract])) OR ("mental disorder"[Title/Abstract])) OR (mental disorders[MeSH Major Topic])) AND ("social media"[Title/Abstract])) OR (social media[MeSH Major Topic])) OR ("social network*"[Title/Abstract])) OR (social networking[MeSH Major Topic])) AND (recruit*[Title/Abstract])) OR (advert*[Title/Abstract])) OR (advertising[MeSH Major Topic]))

Database: SCOPUS
Limitations:

- 2011 – Present
- English

TITLE-ABS (adolescent OR teenager OR youth OR "young adult") AND ("mental health" OR "mental illness" OR "mental disorder") AND ("social media" OR "social network*") AND (recruit* OR advert*)

Database: COCHRANE LIBRARY
Limitations:

- 2011 – Present
- English

TITLE-ABS (adolescent OR teenager OR youth OR "young adult") AND ("mental health" OR "mental illness" OR "mental disorder") AND ("social media" OR "social network*") AND (recruit* OR advert*)

Database: Medline (through EBSCO host)
Limitations:

- 2011 – Present
- English

- All child – 0-18 years

(adolescent OR teenager OR youth OR "young adult") AND ("mental health" OR "mental illness" OR "mental disorder") AND ("social media" OR "social network*") AND (recruit* OR advert*)
