# Supplementary material for: Use of social media in recruiting young people to mental health research: a scoping review
Source: BMJ Open. 2023 Nov 28;13(11):e075290. doi: 10.1136/bmjopen-2023-075290 (PMC10685975; doi:10.1136/bmjopen-2023-075290)
Supplement: Supplementary data [file bmjopen-2023-075290supp002.pdf]

**Supplementary File 2:** Characteristics of the 24 studies included in the scoping review.

| Author, year | Study design | Study aim(s) | Participants | Recruitment methods | Social media platforms | Recruitment related findings |
|---|---|---|---|---|---|---|
| Amon et al., 2022 | Mixed methods exploratory study | To assess the acceptability, safety, user experience, and mental health benefits of a purpose-built, counsellor facilitated social networking service (SNS). | N = 154; young people aged 13-25 (86.4% were between the ages of 13 and 18) | Participants were recruited via the Kids Helpline (KHL) website and referrals from KHL counsellors, as well as through schools and KHL social media channels. | Facebook, Instagram, and Snapchat | Not specified |
| Bauermeister et al., 2022 | Pilot randomised controlled trial | To describe the results of a pilot randomised controlled trial of imi, a web application designed to improve mental health by supporting sexual and gender minority identity affirmation, coping self-efficacy, and coping skill practice. | N = 270; sexual and gender minority youth aged 13 to 19 living in the United States (mean age = 16.49 years) | Paid for social media advertisements | Instagram | Not specified |
| Brawner et al., 2019 | Pilot randomised controlled trial | To estimate the effect of the targeted intervention on consistent condom use, sexual activity, the number of concurrent and sequential sexual partners, and laboratory confirmed HIV/STIs at 3, 6 and 12 month follow up assessments. | N = 108; Black youth aged 14 - 17 years (mean age = 15.8 years) living in Philadelphia, USA | Community-based mental health providers, high schools, community partners (e.g., recreation centres) and provider referrals initially. Due to issues with recruitment they later expanded to include online and social media study promotion, as well as face-to-face recruitment at public venues. | Not specified | Not specified |

| | | | | | | |
|---|---|---|---|---|---|---|
| Chu & Snider, 2013 | Survey | To describe the effectiveness of using Facebook as a recruitment tool for medical research | N = 88; Canadian youth aged 15 - 24 years (mean age = 16.3 years) | An advert highlighting the study was targeted to Facebook members living in Canada aged 15-24 | Facebook | • The average cost per final participant was $15.35.<br>• It was possible to reach a specific population across Canada using social media.<br>• Cost was relatively inexpensive and advertising efforts required minimal personnel.<br>• Facebook's performance tracking allowed optimization of advertising parameters.<br>• The total number of clicks and impressions on weekends was much higher than on weekdays |
| Craig et al., 2023 | Survey | To examine the rates of mental health symptoms of clinical concern and substance use, and assess which COVID-19 related stressors were predictors of these symptoms and substance use in a large Canadian sample of adolescents, with comparisons across genders. | N = 809; mean age = 15.67; adolescents aged 12-18 who lived in Canada. | Recruited through advertisements on social media platforms (Facebook, Instagram) from June 17 to June 31, 2020. | Facebook and Instagram | • Recruiting through social media made targeting adolescents from minority ethnicities and low income and/or remote families without internet or data difficult.<br>• Therefore, the results may not be generalisable to these populations and caution should be taken when interpreting the results. |
| Fitzsimmons-Craft et al., 2021 | Cross sectional survey | To examine exposure (i.e., seeing, following, posting) to body image content emphasizing a thin ideal on various social media platforms and probable ED diagnoses, ED-related quality of life, and psychiatric comorbidities among adolescents and young adult females recruited via social media who endorsed | N = 405; young adult females aged 15 - 25 years (age split by groups 15-17 and 18-25) engaged with pro-ED social media living in the USA | Instagram, Facebook, and Twitter adverts targeting English-speaking individuals in the United States who had demonstrated an interest in and/or followed accounts that were social networking about EDs or ED-related topics. On Reddit, posts were created about the study in two pro-ED related subreddits (i.e., topic-specific communities) | Facebook, Instagram, Twitter, and Reddit | • The greatest proportion of their adolescent sample (15-17 years) were recruited through Instagram at 75%<br>• Participants felt that social media could be used for study recruitment and as a way to link people with treatment. |

| | | | | | | |
|---|---|---|---|---|---|---|
| | | viewing and/or posting pro-ED online content. | | | | |
| Goldbach et al., 2023 | Cross-sectional online survey | To address whether: (1) whether there are differences in behavioural health patterns (i.e., depression, anxiety, and PTSD symptoms) between urban and rural sexual minority adolescents (SMA) and (2) whether these differences are mediated by the reporting of minority stress experiences, using a comprehensive 54-item measure of minority stress designed for use with adolescents. | N = 2,558; SMA in the USA between the ages of 14 and 17; mean age 15.9 | Nationwide targeted paid advertisements through varying social media platforms were used to screen, invite, and enrol SMA into the current study. Respondent driven sampling also occurred. | Not specified (refer to Schrager et al., 2022) | Not specified |
| Kasson et al., 2021 | Feasibility study | To test the feasibility of innovative outreach methods on social media for teens with EDs and to garner feedback from this population to further adapt and tailor a mental health intervention for this population. | The Discovery Group involved N = 14 female teenagers with eating disorders aged 14-17 years; The Testing Group involved N = 30 adolescents with eating disorders aged 14-17 years. | For the Discovery Group phase, Instagram and Facebook were used, specifying keywords related to body image and eating concerns (e.g., weight, shape, thin, waist). For the Testing Group this was then expanded to include Reddit and platforms including Snapchat, TikTok, and YouTube. Advertisements included static images, images with animation, and video advertisements. | Facebook, Instagram, Reddit, TikTok and Snapchat | • Snapchat was found to be the most successful platform for recruitment and Instagram was the second<br>• Platforms such as TikTok and Instagram are more heavily image based and include other features like image filters and video editing that may promote social comparisons and thin ideal |
| Kelleher et al., 2018 | Pilot randomised controlled trial | To determine whether a social media intervention offering resources to young people displaying references to depression appropriately targeted young people with depression and was accessed | N = 25; Tumblr users aged 15-23 (mean age = 17.5 years) who posted about depression using the search term "#depress" | Tumblr messages were sent to eligible individuals. | Tumblr | • Recruitment via Tumblr was feasible<br>• Identifying participants based on depression posts on Tumblr targeted the appropriate population for this study. |

| | | | | | | |
|---|---|---|---|---|---|---|
| | | by, and deemed acceptable by young people. | | | | |
| Kutok et al., 2021 | Feasibility study | To describe the feasibility, cost-effectiveness, and generalisability of a strategy for recruiting adolescents into research studies through social media. | N = 80; Adolescents aged 13 - 17 years (mean age = 15.33 years) from the USA who spoke English; eligibility for the larger study was cybervictimisation and smartphone ownership. | Instagram was used to advertise the study using Facebook Business Manager. Two different ad campaign strategies were used to show the different ads: Reach and Traffic. “Reach” shows an ad to as many users as possible within a target audience. “Traffic” targets users that often click on links within ad. “Story” ads are featured in Instagram stories, which are posts that typically contain a vertical 9:16 ratio video or photo that will disappear after 24 hours. “Feed” ads are featured on Instagram’s regular feed of posts and contain a square video or photo that can produce engagement by being easily liked or shared. | Instagram | • The lowest cost strategy was Traffic campaign + Feed ad placement which was $19 per participant<br>• The most popular title was "Help us learn about online drama" rather than "Tell us about your experience with online drama" and "Stand up against online drama" |
| Lattie et al., 2017 | Feasibility study | To gather information about the feasibility and acceptability of ProjectTECH programme | N = 39; high school students aged 14 - 19 years with depression and substance abuse. | Advertisements on Instagram, through schools and other community settings. | Instagram | • Adolescents responded primarily to social media advertisements.<br>• 28 of the 40 eligible participants were referred to the study through Instagram advertisement. |
| McGuine et al., 2021a | Cross sectional survey | To describe the health of athletes during COVID-19-related school closures and sport cancellations; to assess whether health and wellbeing differed by sex, grade, type of sport(s) played and socioeconomic status. | N = 13002; adolescent athletes from the USA aged 13-19 years (mean age = 16.3 years) | Links to Facebook and Twitter accounts were provided to sports medicine provider colleagues across the United States and to the National Federation of State High School Associations, which passed the links to each USA state high school athletic association. | Facebook and Twitter | • The sample may be biased towards athletes from higher socioeconomic families with easy access to Internet services and social media platforms |

| | | | | | | |
|---|---|---|---|---|---|---|
| McGuine et al., 2021b | Cross sectional survey | To identify changes in the health (mental health, physical activity, and quality of life) of athletes that occurred during the COVID-19 pandemic. | N = 3243 (cohort 1) and N = 5231 (cohort 2); adolescent athletes aged 13 - 19 from Wisconsin, USA | Links to Facebook and Twitter accounts were provided to medical colleagues and the Wisconsin Interscholastic Athletic Association and Wisconsin Athletic Trainers' Association who forwarded the links to high school athletes. | Facebook and Twitter | • The sample may be biased towards athletes from higher socioeconomic families with easy access to Internet services and social media platforms. |
| Mechler et al., 2022 | Randomised clinical trial | To compare the efficacy of IPDT with an established evidence-based treatment (ICBT) for adolescent depression. | N = 272; adolescents in Sweden between the ages of 15 and 19 who had a primary diagnosis of MDD according to the DSM-5 | Participants were recruited nationwide in Sweden through advertisements on social media, as well as contacts with junior and senior high schools, youth associations, social workers, and healthcare providers. | Not specified | Not specified |
| Mitchell et al., 2022 | Survey | To examine how the COVID-19 pandemic may be differentially impacting the well-being of sexual and gender minority (SGM) youth compared with their non-SGM counterparts. | N = 990; youth and emerging adults aged 13 - 23 (age split by groups or 13-17 and 18-23) in the USA, who were English speaking | Study advertisements were placed on social media sites. | Facebook and Instagram | Not specified |
| Morgan et al., 2021 | Survey | To investigate young people's perceived effectiveness of different treatments for mental health problems, the professionals who delivered these, and the experience of negative effects. | N = 557; young people (aged 12 - 25, mean age 18 years) recruited from English-speaking, high-income countries with a current or past mental disorder. | Potential participants were invited to access the study website via a mix of online and offline promotion. Online promotion included links from youth mental health websites. Participants were also recruited via social media and Google ads targeted to searches for help for anxiety and depression. Hard-copy advertisements were distributed to youth mental health clinics and mental health support groups in Australia. | Facebook and Twitter | Not specified |

| | | | | | | |
|---|---|---|---|---|---|---|
| Salk, et al., 2020 | Feasibility study | To introduce and describe the Gender Minority Youth (GMY) study and sample; to provide evidence of the feasibility of using social media recruitment, paired with a waiver of parental consent, to recruit a large and diverse online sample of US transgender and cisgender youth, including multiple subgroups of gender minority youth. | N = 3318; Cisgender and transgender adolescents aged 14-18 years old living in the USA. | Participants were recruited via advertisements on Facebook and Instagram with an advertisement budget of $1500 and a participant incentive budget of $500. Two separate advertisements were used to recruit transgender and cisgender adolescents. All ads included pictures of racially/ethnically diverse adolescents and targeted US users aged 14–18 years. The transgender ad included additional targeting to identify users associated with “interest” labels such as Gender Identity, Genderqueer, and Transgender Activism. | Facebook and Instagram | • Social media recruitment was found to be efficient and inexpensive in reaching adolescents who belong to hidden and stigmatised groups. |
| Santana da Rosa et al., 2019 | Survey | To investigate the influence of the portrayal of suicide in a popular web series (13 Reasons Why) on mood and behaviour | N = 7004; Brazilian adolescents aged 12-18 years (mean age = 14.8) | Participants were recruited through posts on a 13 Reasons Why-themed social media group. | Facebook | Not specified |
| Schrager et al., 2022 | Protocol for a longitudinal component added to the Goldbach (2023) study | To examine how minority stress may change throughout the course of adolescence and how stress trajectories may predict health outcomes. | N = 1,076 (a subset of the original study (35) which involved 2,558 participants); SMA in the USA between the ages of 14 and 17 | Initial participants were recruited through advertising on Facebook/Instagram and YouTube. Advertisements varied slightly by platform, but all included language asking youth to "Share Your Voice" and described basic details of the research study and incentives that participants could earn. Advertising was stratified by gender, geographic region and urbanicity. | Facebook, Instagram, and YouTube | Not specified |
| Smith, et al., 2020 | Survey | To test whether general and minority specific variables are prospectively related to non-suicidal self-injury, suicidal ideation, suicide plans, and | N = 252 sexual and gender minority adolescents aged 14-15 years | Participants were recruited from social media platforms. Study advertisements were posted in forums related to sexual and gender minority populations. The researchers also | Instagram and Tumblr | Not specified |

| | | | | | | |
|---|---|---|---|---|---|---|
| | | suicidal behaviours in sexual and gender minority youth. | | purchased advertisements on social media platforms, using search tags related to SGM identities. | | |
| Stephens et al., 2020 | Randomised controlled trial | To share lessons learned recruiting and enrolling participants via social media into the BRAVE study - a national, multiphase project to design and evaluate a text message and video-based behavioural intervention - and tips to support campaign engagement. | N = 1030; American teenagers and young people aged 15-24 years | We R Native social media channels. Additional recruitment place through listservs associated with tribes, tribal health organisations, Indian education and human service organisations that serve AI/AN teens and young adults. Ads were placed on Facebook and Instagram and were managed using Facebook Ads Manager. Each platform had unique specifications for Ad design, target audience and dates of deployment. "Interests" were used for Ad targeting including location, age, and people who matched specific interests (e.g., National Museum of the American Indian). | Facebook and Instagram | • Ads with a more positive tone typically received greater reach and impressions than posts simply describing the study eligibility criteria. |
| Szlyk et al., 2019 | Cross-sectional online survey | To increase knowledge of barriers to treatment for depression among social media users. | N = 165; US residents aged 15 +, however age was split (15-17 and 18+) who had posted about feeling sad or depressed on social media accounts/groups that post about depression-related topics | A range of social media platforms and online depression forums. Several recruitment methods were used, including private messaging individuals who were networking on depression-focussed groups or who were posting about depression, posting about the study on depression-focused groups, and using advertisements targeted to individuals networking about depression-related topics. | Facebook, Instagram, Twitter, Reddit, and Tumblr | • Findings suggest that social media is a promising platform to recruit individuals with depression symptoms who want mental health support.<br>• Social media has the potential as a setting to engage persons with depression in help-seeking behaviour. |
| Veale et al., 2018 | Survey | To document the prevalence of mental health concerns among transgender youth in Canada and make comparisons with | N = 923; Transgender youth from across Canada aged 14- | Community organisations, healthcare settings, social media, and the researchers' network. | Not specified | Not specified |

| | | | | | | |
|---|---|---|---|---|---|---|
| | | population-based estimates; to explore differences in the prevalence of mental health problems across gender identity subgroups. | 25 years (age was split as 14-18 and 19-25 years) | | | |
| Zimmerman et al., 2018 | Survey | To assess adolescents who watched 13 Reasons Why by asking how it affected them regarding bullying and suicidal ideation. | N = 2,323; Brazilian adolescents aged 15 to 17 | Facebook advertising was used to reach adolescents who liked pages relating to 13 Reasons Why. | Facebook | Not specified |

DSM-5 = Diagnostic and Statistical Manual Version 5; ED = eating disorder; iCBT = internet-based Cognitive Behavioural Therapy; iPDT = internet-based psychodynamic therapy; MDD = major depressive disorder; PTSD = posttraumatic stress disorder; SGM = sexual and gender minority; SMA = sexual minority adolescents.; SNS = social networking service
