# Supplementary material for: Use of social media in recruiting young people to mental health research: a scoping review
Source: BMJ Open. 2023 Nov 28;13(11):e075290. doi: 10.1136/bmjopen-2023-075290 (PMC10685975; doi:10.1136/bmjopen-2023-075290)
Supplement: Author's manuscript [file bmjopen-2023-075290.draft_revisions.pdf]

BMJ Open is committed to open peer review. As part of this commitment we make the peer review history of every article we publish publicly available.

When an article is published we post the peer reviewers' comments and the authors' responses online. We also post the versions of the paper that were used during peer review. These are the versions that the peer review comments apply to.

The versions of the paper that follow are the versions that were submitted during the peer review process. They are not the versions of record or the final published versions. They should not be cited or distributed as the published version of this manuscript.

BMJ Open is an open access journal and the full, final, typeset and author-corrected version of record of the manuscript is available on our site with no access controls, subscription charges or pay-per-view fees (http://bmjopen.bmj.com).

If you have any questions on BMJ Open's open peer review process please email info.bmjopen@bmj.com

BMJ Open

The use of social media in recruiting young people to mental health research: a scoping review

Journal:	*BMJ Open*
Manuscript ID	bmjopen-2023-075290
Article Type:	Original research
Date Submitted by the Author:	03-May-2023

Complete List of Authors:	Smith, Megan; University of Hertfordshire<br>Grohmann, Dominique; University of Hertfordshire, Life and Medical Sciences<br>Trivedi, Daksha; University of Hertfordshire, Health and Social Work

Keywords:	MENTAL HEALTH, Adolescent, Patient Participation, Depression & mood disorders < PSYCHIATRY, PUBLIC HEALTH

SCHOLARONE™
Manuscripts

I, the Submitting Author has the right to grant and does grant on behalf of all authors of the Work (as defined in the below author licence), an exclusive licence and/or a non-exclusive licence for contributions from authors who are: i) UK Crown employees; ii) where BMJ has agreed a CC-BY licence shall apply, and/or iii) in accordance with the terms applicable for US Federal Government officers or employees acting as part of their official duties; on a worldwide, perpetual, irrevocable, royalty-free basis to BMJ Publishing Group Ltd ("BMJ") its licensees and where the relevant Journal is co-owned by BMJ to the co-owners of the Journal, to publish the Work in this journal and any other BMJ products and to exploit all rights, as set out in our licence.

The Submitting Author accepts and understands that any supply made under these terms is made by BMJ to the Submitting Author unless you are acting as an employee on behalf of your employer or a postgraduate student of an affiliated institution which is paying any applicable article publishing charge ("APC") for Open Access articles. Where the Submitting Author wishes to make the Work available on an Open Access basis (and intends to pay the relevant APC), the terms of reuse of such Open Access shall be governed by a Creative Commons licence – details of these licences and which Creative Commons licence will apply to this Work are set out in our licence referred to above.

Other than as permitted in any relevant BMJ Author's Self Archiving Policies, I confirm this Work has not been accepted for publication elsewhere, is not being considered for publication elsewhere and does not duplicate material already published. I confirm all authors consent to publication of this Work and authorise the granting of this licence.

For peer review only

Title: The use of social media in recruiting young people to mental health research: a scoping review

Author names and affiliations: Megan V. A. Smith[1]., Dominique Grohmann[1]., Daksha Trivedi[2]

[1]Centre for Health Services and Clinical Research, University of Hertfordshire, Hertfordshire, Hatfield, United Kingdom
[2]Centre for Research in Public Health and Community Care, University of Hertfordshire, Hatfield, United Kingdom

Corresponding author: Megan Smith; m.smith25@herts.ac.uk

For peer review only

Abstract:

Objectives: This review explored the literature on the use of social media in recruiting young people, aged 13-18, to mental health research. It aimed to identify barriers and facilitators to recruitment and strategies to improve participation in future research.

Design: Scoping review.

Data sources: PubMed, Scopus, Medline (via EBSCO host) and Cochrane Library databases were searched from January 2011 to February 2023.

Eligibility criteria: Studies that outlined social media as a recruitment method and recruited participants between the ages of 13 and 18 years.

Data extraction and synthesis: Data was extracted by two reviewers and cross-checked by a third reviewer. Data on study design, aims, participants, recruitment methods and findings related specifically to social media as a recruitment tool were collected.

Results: 24 journal articles met the inclusion criteria. Studies were predominantly surveys (n=13) conducted in the United States (n=16) recruiting via Facebook (n=16) and/or Instagram (n=14). Only nine of the included articles provided a summary of success and reviewed the efficacy of social media recruitment for young people in mental health research. Type of advertisement, the language used, time of day and the use of keywords were all found to be factors that may influence the success of recruitment through social media, however as these are based on findings from a small number of studies, such potential influences require further investigation.

Conclusion: Social media recruitment can be a successful method for recruiting young people to mental health research. Further research is needed into recruiting socioeconomically marginalised groups using this method, as well as the effectiveness of new social media platforms.

Pre-registration: Open Science Framework (OSF) Registry (https://osf.io/mak75/)

Strengths and limitations of this scoping review

- This is the first scoping review exploring social media platforms for the recruitment of young people to mental health research.
- The review used a rigorous approach to scoping and literature by following the Arksey and O'Malley (2005) five-stage framework.
- The search was conducted on studies between 2011 and 2023 and some studies that are 5-10 years old may not be as relevant now due to how quickly social media platforms change in popularity.
- Studies included were largely conducted in the US, therefore results cannot be generalised to countries where internet devices and social media may not be as easily accessible, or where nature of use is simply different.

1. INTRODUCTION

Mental health conditions are becoming significantly more common among children and young people. The impact of the COVID-19 pandemic on the mental health and wellbeing of children and young people has only added to the global growing concern. In 2022, over 3.5 million 6-23 year olds in England were reported to have a probable or possible mental health disorder (1). Consequently, there has been increasing demand for counselling services, hospital admissions for self-harm and referrals to specialist Child and Adolescent Mental Health Services (CAMHS) in the UK (2). NHS figures have shown record numbers of referrals with the number of young people accessing mental health services reaching a new record of 708,939 in November 2022 (3). Adding to concern, these figures do not account for the large proportion of children and young people not seeking or accessing professional for their mental health conditions (4).

There is a dearth of evidence on the effectiveness of appropriate and timely interventions to improve the mental wellbeing of children and young people (5). Consequently robust, large scale research studies are needed to bridge the gap between supply and demand of mental health treatments. Recruiting eligible and representative participants to research studies, however, can be challenging and is often the most time-consuming aspect of the study process (6,7). Participation in research is generally declining and ineffective recruitment can impact data quality, validity of research findings, and lead to premature termination of the trial (8,9). Recruitment and retention of young people for mental health research is challenging and adds another level of complexity (8,10,11). In particular, participation may be hindered by the stigma of mental illness or fear of negative consequences from self-disclosure (12,13).

Traditional recruitment strategies such as advertisements, telephone calls and mailing letters are often costly, time-consuming and ineffective in representing the target population (14,15). Alternatively, online recruitment strategies are now being adopted to improve enrolment outcomes (16). One example is the use of social media, a group of mobile and internet-based applications allowing users to receive, build and share information worldwide (17). The launch of the social media platforms Myspace and Facebook in 2003 and 2004 respectively, fuelled a growth of online platforms designed to increase social interconnectivity (12). Twitter, Instagram, and Snapchat, among others,

For peer review only

shortly followed and have become an avenue for daily consumption and dissemination of information.

Most young people are active on social networking sites. In 2022 it was reported that 62% of children aged 8-17 had profile(s) on online apps or sites, and using video-sharing platforms such as YouTube or TikTok was the most popular online activity amongst this age group (18). In many cases, those with mental health problems utilise social media platforms to seek support networks and help others (19), indicating that social media could be a successful method of engaging with young people for the purpose of mental health research.

An increasing number of studies are using online recruitment methods and the feasibility of such strategies are being explored. Facebook, in particular has been shown to be a successful recruitment tool for populations who may not respond to traditional recruitment strategies, such as adolescents (20,21). More specifically, the use of paid Facebook advertising, search tool and creation and use of a Facebook Page prove successful in recruiting adolescents to health research (20). Previous reviews that have explored social media platforms in addition to Facebook, found them to be useful for recruitment to mental health research (12,22) however these reviews did not target young people and therefore cannot be generalised to this population.

To date, and to our knowledge, a review has not explored social media platforms for the recruitment of young people to mental health research. The aim of this scoping review is to explore the literature on the use of social media in recruiting young people to mental health research studies and to identify barriers and facilitators to recruitment and strategies to improve participation.

2.0. METHODS

Scoping reviews aim to map all of the relevant literature in a specific area of interest and consequently help to identify any gaps in existing research (23). This approach was adopted as existing knowledge of the literature suggested a lack of previous work on how social media is used to recruit young people to mental health research.

This review utilizes the Arksey and O'Malley framework (23) for scoping reviews which involves five stages; (1) identifying the research question; (2) identifying the relevant studies; (3) study selection; (4) charting the data; (5) collating, summarizing and reporting the results. This scoping review was also conducted in line with the Preferred Reporting Items for Systematic Reviews and Meta Analysis Extension for Scoping Reviews (PRISMA-ScR) (24,25). The methods of this scoping review were pre-registered on the Open Science Framework (OSF) Registry (https://osf.io/mak75/).

2.1. Stage 1: Identifying the research questions

Four research questions directed this scoping review to address current gaps in the literature:

For peer review only

1) What social media platforms are described in the literature for recruiting young people for mental health research?
2) How are social media platforms used to recruit young people for mental health research?
3) What are the barriers and facilitators to recruitment of young people to mental health research using social media?
4) What are the strategies for improving recruitment and participation of young people to mental health research using social media?

2.2. Stage 2: Identifying relevant studies

On the 17th February 2022, searches of the following four databases were carried out: PubMed, Scopus, Medline (via EBSCO host) and Cochrane Library. The search was repeated on 3rd February 2023 to identify relevant papers published since February 2022. Medical subject headings (MeSH) were searched using Boolean operators *"OR/AND"*. The search terms were: (adolescent OR teenager OR youth OR "young adult") AND ("mental health" OR "mental illness*" OR "mental disorder") AND ("social media" OR "social network*") AND (recruit* OR advert*). The search format used in the databases was modified to meet their requirements. Further details regarding the search terms used are provided in Table 1. The final search strategy for PUBMED can be found in supplementary file 1.

Table 1: Search terms

Search term 1	Search term 2	Search term 3	Search term 4
Search operator	*AND*	*AND*	*AND*
Adolescent	"Mental health"	"Social media"	Recruit*
Adolescent	Mental Health	Social media	Advert*
Teenager	"Mental illness*"	"Social network*"	Advertising
Youth	Mental disorders	Social Networking	
"Young adult"	Mental disorder		
Young adult			

MeSH terms [MeSH major topic]

As past reviews in this area have often focused on the use of Facebook (e.g. (16,20) this review aimed to capture publications exploring all social media platforms developed since. As Instagram saw significant growth in December 2010, articles published prior to 1st January 2011 were not included in the database searches, allowing time for any use of Instagram for recruitment to be reported. Therefore, the review years spanned from January 2011 to February 2023.

2.3. Stage 3: Study selection

Studies were included if they (a) outlined the use of social media (e.g., Twitter, Instagram, Facebook, Snapchat and TikTok) as a method to recruit young people aged 13 to 18 and/or those with a mean age of 13-18 (b) addressed a mental ill-health (e.g., depression, anxiety

For peer review only

or eating disorder) (c) were published between January 2011 and February 2023. Studies were excluded from this review if they met any of the following criteria:

1. Did not refer to the use of social media for recruitment of young people aged 13 to 18 in the methodology
2. Were not written in the English language (due to the time and cost involved in translating them to English)
3. Did not address a mental ill-health
4. Were an animal study

Retrieved records from the database searches were extracted and imported into Rayyan, a free web tool created to facilitate literature reviews (26). One reviewer (MS) scanned all imported records and removed duplicates. Titles and abstracts were screened to define the eligibility of each article by two researchers (MS, DG) independently following the aforementioned pre-defined selection criteria. Publications with a title or abstract not meeting the eligibility criteria were excluded. Following the initial screening, full-text articles were reviewed to make a final decision of inclusion. Any discrepancies regarding the inclusion of articles were resolved by discussion and mutual agreement with the third reviewer (DT). The full text of relevant papers was retrieved for further analysis by two reviewers (MS, DG) and was either included or excluded for review based on the eligibility criteria presented below. Primary studies and/or relevant systematic reviews that met the inclusion criteria were included. However, due to the broad age range of two relevant systematic reviews, included articles were individually screened for eligibility. From this, one article met inclusion criteria for the current scoping review and was consequently included in analysis.

The reference lists of all included publications were also examined to ensure all relevant and eligible resources had been identified.

2.4. Stage 4: Charting the data

A data extraction form was developed in Excel by one of the reviewers and agreed by all to determine which variables to extract. Relevant articles were charted using the following column headings:

1. Author(s), year of publication
2. Study design
3. Study aim(s)
4. Participants
5. Recruitment method(s)
6. Social media platforms
7. Recruitment related findings

The two reviewers independently charted the data, discussed the results, and continuously updated the data-charting form in an iterative process.

2.5. Stage 5: Collating, summarizing and reporting the results

For peer review only

All extracted data from the included articles were summarised and tabulated by a member of the research team. In line with Arksey and O'Malley (23), the narrative account is presented in two ways. Firstly, the nature and distribution of the studies included, e.g., study design, country, settings, and participants group were reported. Secondly, the literature was organised according to the following themes which were drawn from the research questions: methods of social media recruitment to recruit young people to mental health research, barriers and facilitators to recruitment of young people to mental health research using social media, how to improve recruitment and participation of young people to mental health research using social media.

3. RESULTS

3.1. Included studies

The searches in 2022 and 2023 are presented together as the same search methodology was adopted. Initial searching of the electronic databases provided 3,669 records. Four hundred and two duplicates were removed leaving 3,267 records to screen for relevance based on title and abstract. Based on title and abstract screening, 3,124 were excluded. One hundred and forty-three full-text articles were assessed by the researchers for inclusion. One hundred and twenty-two were excluded. A total of 21 studies met all inclusion criteria and were subsequently included within this scoping review. When screening the references of the 21 included articles, 2 articles (27,28) were found to fit the inclusion criteria and were therefore also included within the review. As mentioned previously, one paper (29) was included following the screening of two potentially eligible systematic reviews. An overview of the study selection process is provided in Figure 1.

3.2. Study characteristics

Characteristics of the included articles are summarised in Table 2. Study designs included 13 surveys (27,28,37–39,29–36), three pilot randomised controlled trials (40–42), four feasibility studies (43–46), two randomised controlled trials (47,48) and one mixed methods exploratory study (49). One paper (50) presents the protocol of a longitudinal study utilising a subset of Goldbach et al's (33) sample. Schrager et al., (50) provide detailed information of the recruitment methods used by Goldbach et al (33). For the purpose of this review, they are presented as separate studies.

Of the included articles, 16 were conducted in the United States (27,32,43–46,48,50,33–36,38,40–42), three in Canada (29,30,39), one in Australia (49), one in Sweden (47) and one in Brazil (51). One of the included articles recruited participants from both the United States and Brazil (28), and one recruited participants from "major English-speaking countries" including Australia, United States, the United Kingdom and New Zealand (37).

For peer review only

Table 2: Characteristics of the 24 studies included in the scoping review.

Author, year	Study design	Study aim(s)	Participants	Recruitment methods	Social media platforms	Recruitment related findings
Amon et al., 2022	Mixed methods exploratory study	To assess the acceptability, safety, user experience, and mental health benefits of a purpose-built, counsellor facilitated SNS.	N = 154; young people aged 13-25 (86.4% were between the ages of 13 and 18)	Participants were recruited via the Kids Helpline (KHL) website and referrals from KHL counsellors, as well as through schools and KHL social media channels.	Facebook, Instagram, and Snapchat	Not specified
Bauermeister et al., 2022	Pilot randomised controlled trial	To describe the results of a pilot randomised controlled trial of imi, a web application designed to improve mental health by supporting sexual and gender minority identity affirmation, coping self-efficacy, and coping skill practice.	N = 270; sexual and gender minority youth aged 13 to 19 living in the United States (mean age = 16.49 years)	Paid for social media advertisements	Instagram	Not specified
Brawner et al., 2019	Pilot randomised controlled trial	To estimate the effect of the targeted intervention on consistent condom use, sexual activity, the number of concurrent and sequential sexual partners, and laboratory confirmed HIV/STIs at 3, 6 and 12 month follow up assessments.	N = 108; Black youth aged 14 - 17 years (mean age = 15.8 years) living in Philadelphia, USA	Community-based mental health providers, high schools, community partners (e.g., recreation centres) and provider referrals initially. Due to issues with recruitment they later expanded to include online and social media study promotion, as well as face-to-face recruitment at public venues.	Not specified	Not specified

For peer review only

Chu & Snider, 2013	Survey	To describe the effectiveness of using Facebook as a recruitment tool for medical research	N = 88; Canadian youth aged 15 - 24 years (mean age = 16.3 years)	An advert highlighting the study was targeted to Facebook members living in Canada aged 15-24	Facebook	• The average cost per final participant was $15.35.<br>• It was possible to reach a specific population across Canada using social media.<br>• Cost was relatively inexpensive and advertising efforts required minimal personnel.<br>• Facebook's performance tracking allowed optimization of advertising parameters.<br>• The total number of clicks and impressions on weekends was much higher than on weekdays
Craig et al., 2023	Survey	To examine the rates of mental health symptoms of clinical concern and substance use, and assess which COVID-19 related stressors were predictors of these symptoms and substance use in a large Canadian sample of adolescents, with comparisons across genders.	N = 809; mean age = 15.67; adolescents aged 12-18 who lived in Canada.	Recruited through advertisements on social media platforms (Facebook, Instagram) from June 17 to June 31, 2020.	Facebook and Instagram	• Recruiting through social media made targeting adolescents from minority ethnicities and low income and/or remote families without internet or data difficult.<br>• Therefore, the results may not be generalisable to these populations and caution should be taken when interpreting the results.
Fitzsimmons-Craft et al., 2021	Cross sectional survey	To examine exposure (i.e., seeing, following, posting) to body image content emphasizing a thin ideal on various social media platforms and probable ED diagnoses, ED-related quality of life, and psychiatric comorbidities among adolescents and young adult females recruited via social media who endorsed	N = 405; young adult females aged 15 - 25 years (age split by groups 15-17 and 18-25) engaged with pro-ED social media living in the USA	Instagram, Facebook, and Twitter adverts targeting English-speaking individuals in the United States who had demonstrated an interest in and/or followed accounts that were social networking about EDs or ED-related topics. On Reddit, posts were created about the study in two pro-ED related subreddits (i.e., topic-specific communities)	Facebook, Instagram, Twitter, and Reddit	• The greatest proportion of their adolescent sample (15-17 years) were recruited through Instagram at 75%<br>• Participants felt that social media could be used for study recruitment and as a way to link people with treatment.

For peer review only

		viewing and/or posting pro-ED online content.				
Goldbach et al., 2023	Cross-sectional online survey	To address whether: (1) whether there are differences in behavioural health patterns (i.e., depression, anxiety, and PTSD symptoms) between urban and rural sexual minority adolescents (SMA) and (2) whether these differences are mediated by the reporting of minority stress experiences, using a comprehensive 54-item measure of minority stress designed for use with adolescents.	N = 2,558; SMA in the USA between the ages of 14 and 17; mean age 15.9	Nationwide targeted paid advertisements through varying social media platforms were used to screen, invite, and enrol SMA into the current study. Respondent driven sampling also occurred.	Not specified (refer to Schrager et al., 2022)	Not specified
Kasson et al., 2021	Feasibility study	To test the feasibility of innovative outreach methods on social media for teens with EDs and to garner feedback from this population to further adapt and tailor a mental health intervention for this population.	The Discovery Group involved N = 14 female teenagers with eating disorders aged 14-17 years; The Testing Group involved N = 30 adolescents with eating disorders aged 14-17 years.	For the Discovery Group phase, Instagram and Facebook were used, specifying keywords related to body image and eating concerns (e.g., weight, shape, thin, waist). For the Testing Group this was then expanded to include Reddit and platforms including Snapchat, TikTok, and YouTube. Advertisements included static images, images with animation, and video advertisements.	Facebook, Instagram, Reddit, TikTok and Snapchat	• Snapchat was found to be the most successful platform for recruitment and Instagram was the second<br>• Platforms such as TikTok and Instagram are more heavily image based and include other features like image filters and video editing that may promote social comparisons and thin ideal
Kelleher et al., 2018	Pilot randomised controlled trial	To determine whether a social media intervention offering resources to young people displaying references to depression appropriately targeted young people with depression and was accessed	N = 25; Tumblr users aged 15-23 (mean age = 17.5 years) who posted about depression using the search term "#depress"	Tumblr messages were sent to eligible individuals.	Tumblr	• Recruitment via Tumblr was feasible<br>• Identifying participants based on depression posts on Tumblr targeted the appropriate population for this study.

For peer review only

		by, and deemed acceptable by young people.				
Kutok et al., 2021	Feasibility study	To describe the feasibility, cost-effectiveness, and generalisability of a strategy for recruiting adolescents into research studies through social media.	N = 80; Adolescents aged 13 - 17 years (mean age = 15.33 years) from the USA who spoke English; eligibility for the larger study was cybervictimisation and smartphone ownership.	Instagram was used to advertise the study using Facebook Business Manager. Two different ad campaign strategies were used to show the different ads: Reach and Traffic. "Reach" shows an ad to as many users as possible within a target audience. "Traffic" targets users that often click on links within ad. "Story" ads are featured in Instagram stories, which are posts that typically contain a vertical 9:16 ratio video or photo that will disappear after 24 hours. "Feed" ads are featured on Instagram's regular feed of posts and contain a square video or photo that can produce engagement by being easily liked or shared.	Instagram	• The lowest cost strategy was Traffic campaign + Feed ad placement which was $19 per participant<br>• The most popular title was "Help us learn about online drama" rather than "Tell us about your experience with online drama" and "Stand up against online drama"
Lattie et al., 2017	Feasibility study	To gather information about the feasibility and acceptability of ProjectTECH programme	N = 39; high school students aged 14 - 19 years with depression and substance abuse.	Advertisements on Instagram, through schools and other community settings.	Instagram	• Adolescents responded primarily to social media advertisements.<br>• 28 of the 40 eligible participants were referred to the study through Instagram advertisement.
McGuine et al., 2021a	Cross sectional survey	To describe the health of athletes during COVID-19-related school closures and sport cancellations; to assess whether health and wellbeing differed by sex, grade, type of sport(s) played and socioeconomic status.	N = 13002; adolescent athletes from the USA aged 13-19 years (mean age = 16.3 years)	Links to Facebook and Twitter accounts were provided to sports medicine provider colleagues across the United States and to the National Federation of State High School Associations, which passed the links to each USA state high school athletic association.	Facebook and Twitter	• The sample may be biased towards athletes from higher socioeconomic families with easy access to Internet services and social media platforms

For peer review only

McGuine et al., 2021b	Cross sectional survey	To identify changes in the health (mental health, physical activity, and quality of life) of athletes that occurred during the COVID-19 pandemic.	N = 3243 (cohort 1) and N = 5231 (cohort 2); adolescent athletes aged 13 - 19 from Wisconsin, USA	Links to Facebook and Twitter accounts were provided to medical colleagues and the Wisconsin Interscholastic Athletic Association and Wisconsin Athletic Trainers' Association who forwarded the links to high school athletes.	Facebook and Twitter	• The sample may be biased towards athletes from higher socioeconomic families with easy access to Internet services and social media platforms.
Mechler et al., 2022	Randomised clinical trial	To compare the efficacy of IPDT with an established evidence-based treatment (ICBT) for adolescent depression.	N = 272; adolescents in Sweden between the ages of 15 and 19 who had a primary diagnosis of MDD according to the DSM-5	Participants were recruited nationwide in Sweden through advertisements on social media, as well as contacts with junior and senior high schools, youth associations, social workers, and healthcare providers.	Not specified	Not specified
Mitchell et al., 2022	Survey	To examine how the COVID-19 pandemic may be differentially impacting the well-being of sexual and gender minority (SGM) youth compared with their non-SGM counterparts.	N = 990; youth and emerging adults aged 13 - 23 (age split by groups or 13-17 and 18-23) in the USA, who were English speaking	Study advertisements were placed on social media sites.	Facebook and Instagram	Not specified
Morgan et al., 2021	Survey	To investigate young people's perceived effectiveness of different treatments for mental health problems, the professionals who delivered these, and the experience of negative effects.	N = 557; young people (aged 12 - 25, mean age 18 years) recruited from English-speaking, high-income countries with a current or past mental disorder.	Potential participants were invited to access the study website via a mix of online and offline promotion. Online promotion included links from youth mental health websites. Participants were also recruited via social media and Google ads targeted to searches for help for anxiety and depression. Hard-copy advertisements were distributed to youth mental health clinics and mental health support groups in Australia.	Facebook and Twitter	Not specified

For peer review only

Salk, et al., 2020	Feasibility study	To introduce and describe the Gender Minority Youth (GMY) study and sample; to provide evidence of the feasibility of using social media recruitment, paired with a waiver of parental consent, to recruit a large and diverse online sample of US transgender and cisgender youth, including multiple subgroups of gender minority youth.	N = 3318; Cisgender and transgender adolescents aged 14-18 years old living in the USA.	Participants were recruited via advertisements on Facebook and Instagram with an advertisement budget of $1500 and a participant incentive budget of $500. Two separate advertisements were used to recruit transgender and cisgender adolescents. All ads included pictures of racially/ethnically diverse adolescents and targeted US users aged 14–18 years. The transgender ad included additional targeting to identify users associated with "interest" labels such as Gender Identity, Genderqueer, and Transgender Activism.	Facebook and Instagram	• Social media recruitment was found to be efficient and inexpensive in reaching adolescents who belong to hidden and stigmatised groups.
Santana da Rosa et al., 2019	Survey	To investigate the influence of the portrayal of suicide in a popular web series (13 Reasons Why) on mood and behaviour	N = 7004; Brazilian adolescents aged 12-18 years (mean age = 14.8)	Participants were recruited through posts on a 13 Reasons Why-themed social media group.	Facebook	Not specified
Schrager et al., 2022	Protocol for a longitudinal component added to the Goldbach (2023) study	To examine how minority stress may change throughout the course of adolescence and how stress trajectories may predict health outcomes.	N = 1,076 (a subset of the original study (Goldbach et al., 2023) which involved 2,558 participants); SMA in the USA between the ages of 14 and 17	Initial participants were recruited through advertising on Facebook/Instagram and YouTube. Advertisements varied slightly by platform, but all included language asking youth to "Share Your Voice" and described basic details of the research study and incentives that participants could earn. Advertising was stratified by gender, geographic region and urbanicity.	Facebook, Instagram, and YouTube	Not specified
Smith, et al., 2020	Survey	To test whether general and minority specific variables are prospectively related to non-suicidal self-injury, suicidal ideation, suicide plans, and	N = 252 sexual and gender minority adolescents aged 14-15 years	Participants were recruited from social media platforms. Study advertisements were posted in forums related to sexual and gender minority populations. The researchers also	Instagram and Tumblr	Not specified

		suicidal behaviours in sexual and gender minority youth.		purchased advertisements on social media platforms, using search tags related to SGM identities.		
Stephens et al., 2020	Randomised controlled trial	To share lessons learned recruiting and enrolling participants via social media into the BRAVE study - a national, multiphase project to design and evaluate a text message and video-based behavioural intervention - and tips to support campaign engagement.	N = 1030; American teenagers and young people aged 15-24 years	We R Native social media channels. Additional recruitment place through listservs associated with tribes, tribal health organisations, Indian education and human service organisations that serve AI/AN teens and young adults. Ads were placed on Facebook and Instagram and were managed using Facebook Ads Manager. Each platform had unique specifications for Ad design, target audience and dates of deployment. "Interests" were used for Ad targeting including location, age, and people who matched specific interests (e.g., National Museum of the American Indian).	Facebook and Instagram	• Ads with a more positive tone typically received greater reach and impressions than posts simply describing the study eligibility criteria.
Szlyk et al., 2019	Cross-sectional online survey	To increase knowledge of barriers to treatment for depression among social media users.	N = 165; US residents aged 15 +, however age was split (15-17 and 18+) who had posted about feeling sad or depressed on social media accounts/groups that post about depression-related topics	A range of social media platforms and online depression forums. Several recruitment methods were used, including private messaging individuals who were networking on depression-focussed groups or who were posting about depression, posting about the study on depression-focused groups, and using advertisements targeted to individuals networking about depression-related topics.	Facebook, Instagram, Twitter, Reddit, and Tumblr	• Findings suggest that social media is a promising platform to recruit individuals with depression symptoms who want mental health support. • Social media has the potential as a setting to engage persons with depression in help-seeking behaviour.
Veale et al., 2018	Survey	To document the prevalence of mental health concerns among transgender youth in Canada and make comparisons with	N = 923; Transgender youth from across Canada aged 14-	Community organisations, healthcare settings, social media, and the researchers' network.	Not specified	Not specified

For peer review only

		population-based estimates; to explore differences in the prevalence of mental health problems across gender identity subgroups.	25 years (age was split as 14-18 and 19-25 years)			
Zimmerman et al., 2018	Survey	To assess adolescents who watched 13 Reasons Why by asking how it affected them regarding bullying and suicidal ideation.	N = 2,323; Brazilian adolescents aged 15 to 17	Facebook advertising was used to reach adolescents who liked pages relating to 13 Reasons Why.	Facebook	Not specified

DSM-5 = Diagnostic and Statistical Manual Version 5; ED = eating disorder; iCBT = internet-based Cognitive Behavioural Therapy; iPDT = internet-based psychodynamic therapy; MDD = major depressive disorder; PTSD = posttraumatic stress disorder; SGM = sexual and gender minority; SMA = sexual minority adolescents; SNS = social networking service

For peer review only

3.3. Participants

Some articles grouped their entire sample by age (32,36,38,39), therefore only the data from the groups that were eligible for this review were considered (13–18-year-olds). Four studies recruited youth with a mental health condition (37,43,45,47) and two recruited athletes (34,35). Nine studies recruited adolescents from specific demographic backgrounds e.g., sexual and gender minorities (27,33,39,42,46,50), America Indian and Alaska Native (AI/AN) youth (48) and heterosexually active Black youth (40). Four studies recruited youth based on social media activity i.e. posting online about being sad or depressed (38,41), females engaging in pro-eating disorder posts (32), individuals who had liked online social media pages for the television series “13 Reasons Why” (28,31). One study recruited youth who had been affected by violence (29) and one recruited youth seeking support for issues related to family discord and associated impacts on emotional well-being (49). The remaining three studies recruited youth with no particular characteristics noted (36,44,52).

3.4. Methods of social media recruitment

Among all retrieved articles, 20 described at least one specific type of social media as a method of recruitment (see Table 2 for distribution). Facebook was used most commonly by 16 of the 24 included articles (28,29,38,43,46,48–50,30–37), 14 used Instagram (27,30,46,48–50,32,33,36,38,42–45) and 5 used Twitter (32,34,35,37,38). Other, less commonly used social media platforms were Reddit (32,38,43), Tumblr (28,38,41), Snapchat (43,49), TikTok (43) and YouTube (33,43,50). Three articles, although discussing the use of social media for recruitment, did not specify the platforms used (39,40,47).

Of the articles which specified the platforms used, 12 of these used more than one social media platform to advertise (27,32,48,50,33–38,43,46). Half of the articles (n=12) used advertisements tailored to reach their target population by setting restrictions, for example keywords, age, interests or location (27,28,48,50,32,33,36–38,43,44,46). Three articles specified using Facebook Business Manager to manage Instagram and/or Facebook advertisements (43,44,48).

Some articles used a combination of online and offline advertisement methods. Brawner et al., (40) for example, initially aimed to recruit through community-based mental health providers, high schools, community partners (e.g., recreation centres) and provider referrals, however, this was not successful so introduced social media study promotion. Lattie et al., (45) advertised via Instagram as well as offline e.g., through schools, community settings, fliers, university databases and at conferences. Morgan et al., (37) also used a combination of paid advertisements on Facebook and Google as well as hard copy advertisements in youth mental health clinics and support groups. Amon et al., (49) recruited specifically through the Kids Helpline (KHL) website, referrals from KHL counsellors, and schools, as well as through social media. Goldbach et al. (33) and Schrager et al. (50) used social media advertising along with respondent-driven sampling, where participants could earn gift cards through referring eligible participants. Mechler et al., (47) also recruited through contacts with schools, youth associations, social workers and healthcare providers.

For peer review only

3.5. Barriers to social media recruitment

Few articles discussed barriers to social media recruitment. One potential barrier highlighted was that this method of recruitment may be more bias to individuals with a higher socioeconomic status who have access to internet facilities and therefore likely to be active on social media (29,30,34,35) potentially leading to an unrepresentative sample. Cost may be seen as a potential barrier, and several studies revealed their total cost of using social media advertising: $1,351 (29); $1,536 (46); $1,591 (44); over $5,000 (48). One study highlighted the potential complexity of using social media advertising, stating that using Facebook Business Manager to monitor their advert statistics could be complicated (44).

3.6. Facilitators of social media recruitment

Six of the 24 included articles discussed facilitators of social media recruitment in detail. Regarding most effective type of social media platform for recruitment, (43) found Snapchat to be the most successful, with Instagram as the second most successful in a comparison of five social media sites. (32) also found Instagram to be highly effective at recruiting their adolescent age group (15–17-year-olds) with 76% of the sample recruited through this platform. One study (44) compared combinations of Instagram advertisements and found that those with the campaign strategy “Traffic” (targeting users that often click on links within an ad) and the advertisement placement “Feed” (on Instagram’s regular feed of posts, as opposed to a “Story” which disappears after 24 hours) were the most effective combination in terms of recruitment success and cost-effectiveness. Regarding wording of social media advertisements, two studies found that those with a more positive tone or those that suggested helping the researchers received more interest (44,48). Chu and Snider (29) investigated Facebook advertising throughout the week and found the number of clicks and impressions were much higher on weekends than weekdays. One study by Kelleher et al (41) successfully recruited youth through Tumblr who had posted about depression, through searching #depress to identify potentially suitable individuals. Several studies made use of “labels” or “keywords” in order to target a certain population (32,43,46), however the effectiveness of this strategy in aiding recruitment is difficult to determine as there were no comparator advertisements.

3.7. How to improve recruitment and participation of young people to mental health research using social media

Few articles addressed methods to improve recruitment of this population group using social media. Several studies found that advertising through Instagram was beneficial in their recruitment, which suggests that it may be a suitable platform in attracting young people to mental health research (32,43). Snapchat was also found to be highly effective at recruiting (43). Both of these social media platforms are heavily image based which may have led to the increase in interest and is a factor to consider in future research. Kutok et al (44) noted that social media is an ever-changing environment and therefore highlighted the importance of being flexible. For example, Instagram and Snapchat may be effective currently, however in several years’ time other platforms may be more popular and therefore researchers should be open to trying a range of platforms. Several studies found that adverts with a more positive tone received more interest (44,48), which suggests that

For peer review only

the language used when advertising the study should be an important consideration for future studies.

4. DISCUSSION

4.1. Main findings

The aim of this scoping review was to explore the literature on the use of social media in recruiting young people to mental health research and to identify barriers and facilitators to recruitment and strategies for improving recruitment. From the 3,308 articles identified through the four databases, 24 studies met the inclusion criteria.

The included studies investigated a range of populations recruited successfully, suggesting that social media may be a suitable method for engaging with and recruiting specific populations. This is consistent with previous reviews investigating social media as a recruitment method for health research (53,54). However, further research is required to determine the suitability of digital delivery to socioeconomically and digitally marginalised youth to mental health research (55). This is even more crucial considering socioeconomically marginalised, and consequently digitally excluded, youth are more likely to develop mental health problems than more socioeconomically advantaged peers (55–57).

Regarding type of social media platform, the majority of studies used the platform Facebook, consistent with previous reviews focusing on the general population (20,21). Facebook use in 2014-2015 amongst teens was at 71%, which has reduced considerably to 32% in 2021 (58). This decrease in popularity could imply that other social media platforms may be preferred when targeting this population and highlights the importance of keeping up to date with the most popular platforms with this age group at the time of recruitment. Instagram was also a popular platform of choice. Kasson and colleagues (43) found Snapchat and Instagram to be the most effective social media sites regarding percentage of the sample recruited. They suggested that the nature of these sites being heavily image based may attract this population compared with other networking sites. However, few of the studies compared effectiveness across a range of platforms, therefore these findings should be interpreted with caution and comparison of sites is something that should be investigated further in the future.

Regarding barriers to using social media as a recruitment technique, one of the main concerns is the potential cost of advertising, with included studies ranging from $1,351 to over $5,000. Affordability will largely depend on decisions made by the research team and the funding that has been allocated, however this highlights a need for future studies to investigate cost-effectiveness of social media compared to other forms of recruitment to determine which is more appropriate, particularly if teams have a limited budget. Another potential barrier to using social media for recruitment is the potential complexity of advertising sites, however this is likely to vary depending on the research team's knowledge and experience in this area. An additional barrier and potential consideration are the caveats of minimum age requirements to sign up to social networking sites, as well as specific considerations around consent and confidentiality. This must not be overlooked if social media are to be used in adolescent research (20).

For peer review only

4.2. Strengths and limitations

To our knowledge, this is the first scoping review exploring all social media platforms for the recruitment of young people to mental health research. Furthermore, it followed the rigorous Arksey and O'Malley (2005) five-stage framework which allowed for transparency. To appreciate the findings of this review the following limitations should be acknowledged. Firstly, the search was conducted from 2011 to 2023 and due to the ever-changing nature of popularity of social media sites, some of the studies that were conducted several years ago may not be as relevant now. Another limitation is that most of the studies included were from Western countries such as Canada and the United States, therefore the results cannot be generalised to countries in which social media use is restricted or different from these countries. Some of the social media platforms investigated here are banned in countries including Iran, China and Uganda, therefore results relating to certain platforms may not be applicable. Finally, only a few of the papers included reported data on the specifics of using social media as a recruitment method, for example the days that are best to advertise, tone of advert and any keywords used, therefore these findings should be interpreted with caution.

4.3. Future suggestions

Although the findings and reflections from these papers may be of help for other researchers who are looking to recruit using social media, many of those included did not evaluate the effectiveness of social media as a recruitment technique. Therefore, we suggest that future studies provide more detail and report on the effectiveness of various strategies adopted such as the platform, type of advertisement and language used, as well as the number of participants recruited through social media compared to more traditional recruitment methods if these are also adopted. Additionally, it would be beneficial for researchers to investigate the cost-effectiveness of social media compared to other types of recruitment to ascertain whether it is a cost-effective method. Such findings may then contribute towards developing a guidance document on this topic that can be shared amongst researchers to increase knowledge and awareness when considering this recruitment method for their research.

5. CONCLUSIONS

Recruiting young people to mental health research can be challenging and increasingly, online recruitment methods are being used to increase enrolment and accessibility to participants. This review concludes that social media can be a successful method for recruiting young people to mental health research in terms of reaching the target number of participants, recruiting specific populations and in some cases be more successful compared with traditional methods of recruitment. Further research is needed into recruiting socioeconomically marginalised groups using this method, as well as the effectiveness of new social media platforms. As technology continues to advance, so must recruitment methods.

For peer review only

Figures Figure 1: PRISMA Flow Diagram

Acknowledgments The authors would like to thank Lauren Denyer for her assistance in developing the protocol for this scoping review.

Author contributions MS and DG contributed to the conception and design of this review. MS conducted the search and selection process. MS and DG extracted all data. DT provided advice and guidance on the analysis and interpretation of results. MS and DG produced the first draft of the manuscript. All authors contributed to writing and approved the final draft of the manuscript.

Competing interests None declared.

Funding This work was supported by the Health Technology Assessment funding stream of the National Institutes of Health Research (NIHRA) (Reference Number: 17/78/10).

For peer review only

REFERENCES

1. Young Minds. Impact Report [Internet]. 2023. Available from: https://www.youngminds.org.uk/about-us/reports-and-impact/impact-report-2022/highlight-tour/

2. Pitchforth J, Fahy K, Ford T, Wolpert M, Viner RM, Hargreaves DS. Mental health and well-being trends among children and young people in the UK, 1995-2014: analysis of repeated cross-sectional national health surveys. Psychol Med. 2019 Jun;49(8):1275–85.

3. NHS Digital. Mental Health Services Monthly Statistics [Internet]. 2023. Available from: https://digital.nhs.uk/data-and-information/data-collections-and-data-sets/data-sets/mental-health-services-data-set/statistics-and-reports

4. Radez J, Reardon T, Creswell C, Lawrence PJ, Evdoka-Burton G, Waite P. Why do children and adolescents (not) seek and access professional help for their mental health problems? A systematic review of quantitative and qualitative studies. Eur Child Adolesc Psychiatry [Internet]. 2021;30(2):183–211. Available from: https://doi.org/10.1007/s00787-019-01469-4

5. Schley C, Pace N, Mann R, McKenzie C, McRoberts A, Parker A. The headspace Brief Interventions Clinic: Increasing timely access to effective treatments for young people with early signs of mental health problems. Early Interv Psychiatry. 2019;13(5):1073–82.

6. Orri M, Lipset CH, Jacobs BP, Costello AJ, Cummings SR. Web-based trial to evaluate the efficacy and safety of tolterodine ER 4 mg in participants with overactive bladder: REMOTE trial. Contemp Clin Trials. 2014;38(2):190–7.

7. Moseson H, Kumar S, Juusola JL. Comparison of study samples recruited with virtual versus traditional recruitment methods. Contemp Clin trials Commun. 2020;19:100590.

8. Hoffmann SH, Folker AP, Buskbjerg M, Folker MP, Jezek AH, Svarta DL, et al. Potential of Online Recruitment Among 15-25-Year Olds: Feasibility Randomized Controlled Trial. JMIR Form Res. 2022;6(5):e35874.

9. Bull J, Uhlenbrauck G, Mahon E, Furlong P, Roberts J. Barriers to Trial Recruitment and Possible Solutions. Appl Clin Trials. 2016;25(2).

10. Moreno MA, Waite A, Pumper M, Colburn T, Holm M, Mendoza J. Recruiting Adolescent Research Participants: In-Person Compared to Social Media Approaches. Cyberpsychol Behav Soc Netw. 2017 Jan;20(1):64–7.

11. Liu Y, Pencheon E, Hunter RM, Moncrieff J, Freemantle N. Recruitment and retention strategies in mental health trials–a systematic review. PLoS One. 2018;13(8):e0203127.

12. Sanchez C, Grzenda A, Varias A, Widge AS, Carpenter LL, McDonald WM, et al. Social media recruitment for mental health research: a systematic review. Compr Psychiatry. 2020;103:152197.

13. Woodall A, Morgan C, Sloan C, Howard L. Barriers to participation in mental health research: are there specific gender, ethnicity and age related barriers? BMC

For peer review only

Psychiatry. 2010;10(1):1–10.

14. Surdam J, Daly B, Fulton S, Margevisius S, Schluchter M, Flocke S, et al. Recruitment strategies for nurse enrollment in an online study. Nurs Res. 2020;69(1):69.

15. Bethel C, Rainbow JG, Dudding KM. Recruiting nurses via social media for survey studies. Nurs Res. 2021;70(3):231–5.

16. Thornton L, Batterham PJ, Fassnacht DB, Kay-Lambkin F, Calear AL, Hunt S. Recruiting for health, medical or psychosocial research using Facebook: Systematic review. Internet Interv. 2016;4:72–81.

17. Cataldo I, Lepri B, Neoh MJY, Esposito G. Social media usage and development of psychiatric disorders in childhood and adolescence: a review. Front psychiatry. 2021;11:1332.

18. Ofcom. Children and parents: media use and attitudes report 2022 [Internet]. 2022. Available from: https://www.ofcom.org.uk/__data/assets/pdf_file/0024/234609/childrens-media-use-and-attitudes-report-2022.pdf

19. Naslund JA, Aschbrenner KA, Marsch LA, Bartels SJ. The future of mental health care: peer-to-peer support and social media. 2045; Available from: https://doi.org/10.1017/S2045796015001067

20. Amon KL, Campbell AJ, Hawke C, Steinbeck K. Facebook as a recruitment tool for adolescent health research: a systematic review. Acad Pediatr. 2014;14(5):439-447.e4.

21. Amon KL, Paxton K, Klineberg E, Riley L, Hawke C, Steinbeck K. Insights into Facebook Pages: an early adolescent health research study page targeted at parents. Int J Adolesc Med Health. 2016 Feb;28(1):69–77.

22. Bour C, Ahne A, Schmitz S, Perchoux C, Dessenne C, Fagherazzi G. The use of social media for health research purposes: scoping review. J Med Internet Res. 2021;23(5):e25736.

23. Arksey H, O'Malley L. Scoping studies: towards a methodological framework. https://doi.org/101080/1364557032000119616 [Internet]. 2007 Feb [cited 2022 Sep 22];8(1):19–32. Available from: https://www.tandfonline.com/doi/abs/10.1080/1364557032000119616

24. Tricco AC, Lillie E, Zarin W, O'Brien KK, Colquhoun H, Levac D, et al. PRISMA extension for scoping reviews (PRISMA-ScR): checklist and explanation. Ann Intern Med. 2018;169(7):467–73.

25. Peters MDJ, Marnie C, Tricco AC, Pollock D, Munn Z, Alexander L, et al. Updated methodological guidance for the conduct of scoping reviews. JBI Evid Synth [Internet]. 2020;18(10). Available from: https://journals.lww.com/jbisrir/Fulltext/2020/10000/Updated_methodological_guidance_for_the_conduct_of.4.aspx

26. Ouzzani M, Hammady H, Fedorowicz Z, Elmagarmid A. Rayyan---a web and mobile app for systematic reviews. Syst Rev [Internet]. 2016;5(1):210. Available from: http://dx.doi.org/10.1186/s13643-016-0384-4

For peer review only

27. Smith DM, Wang SB, Carter ML, Fox KR, Hooley JM. Longitudinal predictors of self-injurious thoughts and behaviors in sexual and gender minority adolescents. J Abnorm Psychol. 2020;129(1):114.

28. Zimerman A, Caye A, Salum GA, Passos IC, Kieling C. Revisiting the Werther effect in the 21st century: bullying and suicidality among adolescents who watched 13 Reasons Why. J Am Acad Child Adolesc Psychiatry. 2018;57(8):610–3.

29. Chu JL, Snider CE. Use of a social networking web site for recruiting Canadian youth for medical research. J Adolesc Heal Off Publ Soc Adolesc Med. 2013 Jun;52(6):792–4.

30. Craig SG, Ames ME, Bondi BC, Pepler DJ. Canadian adolescents' mental health and substance use during the COVID-19 pandemic: Associations with COVID-19 stressors. Vol. 55, Canadian Journal of Behavioural Science / Revue canadienne des sciences du comportement. Craig, Stephanie G.: Department of Psychology, York University, 4700 Keele Street, Toronto, ON, Canada, M3J 1P3, sgcraig@yorku.ca: Educational Publishing Foundation; 2023. p. 46–55.

31. da Rosa GS, Andrades GS, Caye A, Hidalgo MP, de Oliveira MAB, Pilz LK. Thirteen Reasons Why: The impact of suicide portrayal on adolescents' mental health. J Psychiatr Res. 2019;108:2–6.

32. Fitzsimmons-Craft EE, Krauss MJ, Costello SJ, Floyd GM, Wilfley DE, Cavazos-Rehg PA. Adolescents and young adults engaged with pro-eating disorder social media: eating disorder and comorbid psychopathology, health care utilization, treatment barriers, and opinions on harnessing technology for treatment. Eat Weight Disord. 2020 Dec;25(6):1681–92.

33. Goldbach JT, Parra LA, O'Brien RP, Rhoades H, Schrager SM. Explaining behavioral health differences in urban and rural sexual minority adolescents: A longitudinal investigation of minority stress in a diverse national sample of sexual minority adolescents: A longitudinal investigation of minority stress in a div. J Rural Heal Off J Am Rural Heal Assoc Natl Rural Heal Care Assoc. 2023 Jan;39(1):262–71.

34. McGuine TA, Biese KM, Petrovska L, Hetzel SJ, Reardon CL, Kliethermes S, et al. Changes in the Health of Adolescent Athletes: A Comparison of Health Measures Collected before and during the COVID-19 Pandemic. J Athl Train. 2021;56(8):836–44.

35. McGuine TA, Biese KM, Petrovska L, Hetzel SJ, Reardon C, Kliethermes S, et al. Mental Health, Physical Activity, and Quality of Life of US Adolescent Athletes During COVID-19-Related School Closures and Sport Cancellations: A Study of 13 000 Athletes. J Athl Train. 2021 Jan;56(1):11–9.

36. Mitchell KJ, Ybarra ML, Banyard V, Goodman KL, Jones LM. Impact of the COVID-19 Pandemic on Perceptions of Health and Well-Being Among Sexual and Gender Minority Adolescents and Emerging Adults. LGBT Heal. 2022 Jan;9(1):34–42.

37. Morgan AJ, Ross AM, Yap MBH, Reavley NJ, Parker A, Simmons MB, et al. What works for mental health problems in youth? Survey of real-world experiences of treatments and side effects. Early Interv Psychiatry. 2021;15(6):1502–12.

38. Szlyk H, Deng J, Xu C, Krauss MJ, Cavazos-Rehg PA. Leveraging social media to explore the barriers to treatment among individuals with depressive symptoms. Depress

For peer review only

Anxiety [Internet]. 2020;37(5):458 – 465. Available from: https://www.scopus.com/inward/record.uri?eid=2-s2.0-85077903892&doi=10.1002%2Fda.22990&partnerID=40&md5=475bbd981c9ef52a9c2a2fb19bef010d

39. Veale JF, Watson RJ, Peter T, Saewyc EM. Mental Health Disparities Among Canadian Transgender Youth. J Adolesc Heal Off Publ Soc Adolesc Med. 2017 Jan;60(1):44–9.

40. Brawner BM, Jemmott LS, Hanlon AL, Lozano AJ, Abboud S, Ahmed C, et al. Results from Project GOLD: A pilot randomized controlled trial of a psychoeducational HIV/STI prevention intervention for black youth. AIDS Care. 2021 Jun;33(6):767–85.

41. Kelleher E, Moreno M, Wilt MP. Recruitment of participants and delivery of online mental health resources for depressed individuals using tumblr: Pilot randomized control trial. JMIR Res Protoc [Internet]. 2018;7(4). Available from: https://www.scopus.com/inward/record.uri?eid=2-s2.0-85081410138&doi=10.2196%2Fresprot.9421&partnerID=40&md5=7775ffd7c27cd89f6ef4c9f59575d5a0

42. Bauermeister J, Choi SK, Bruehlman-Senecal E, Golinkoff J, Taboada A, Lavra J, et al. An Identity-Affirming Web Application to Help Sexual and Gender Minority Youth Cope With Minority Stress: Pilot Randomized Controlled Trial. J Med Internet Res [Internet]. 2022 Aug 1 [cited 2023 Mar 29];24(8). Available from: https://pubmed.ncbi.nlm.nih.gov/35916700/

43. Kasson E, Vázquez MM, Doroshenko C, Fitzsimmons-Craft EE, Wilfley DE, Taylor CB, et al. Exploring Social Media Recruitment Strategies and Preliminary Acceptability of an mHealth Tool for Teens with Eating Disorders. Int J Environ Res Public Health. 2021 Jul;18(15).

44. Kutok ER, Doria N, Dunsiger S, Patena J V, Nugent NR, Riese A, et al. Feasibility and Cost of Using Instagram to Recruit Adolescents to a Remote Intervention. J Adolesc Heal Off Publ Soc Adolesc Med. 2021 Nov;69(5):838–46.

45. Lattie EG, Ho J, Sargent E, Tomasino KN, Smith JD, Brown CH, et al. Teens engaged in collaborative health: The feasibility and acceptability of an online skill-building intervention for adolescents at risk for depression. Internet Interv [Internet]. 2017;8:15 – 26. Available from: https://www.scopus.com/inward/record.uri?eid=2-s2.0-85014559187&doi=10.1016%2Fj.invent.2017.02.003&partnerID=40&md5=ca5264fed2384526cf9e3bf57f35fe90

46. Salk RH, Thoma BC, Choukas-Bradley S. The Gender Minority Youth Study: Overview of Methods and Social Media Recruitment of a Nationwide Sample of U.S. Cisgender and Transgender Adolescents. Arch Sex Behav. 2020 Oct;49(7):2601–10.

47. Mechler J, Lindqvist K, Carlbring P, Topooco N, Falkenström F, Lilliengren P, et al. Therapist-guided internet-based psychodynamic therapy versus cognitive behavioural therapy for adolescent depression in Sweden: a randomised, clinical, non-inferiority trial. Lancet Digit Heal. 2022 Aug 1;4(8):e594–603.

48. Stephens D, Peterson R, Singer M, Johnson J, Rushing SC, Kelley A. Recruiting and Engaging American Indian and Alaska Native Teens and Young Adults in a SMS Help-Seeking Intervention: Lessons Learned from the BRAVE Study. Int J Environ Res Public

For peer review only

Health. 2020 Dec;17(24).

49. Amon K, Ridout B, Forsyth R, Campbell A. Online Group Counseling for Young People Through a Customized Social Networking Platform: Phase 2 of Kids Helpline Circles. https://home.liebertpub.com/cyber [Internet]. 2022 Sep 14 [cited 2023 Mar 29];25(9):580–8. Available from: https://www.liebertpub.com/doi/10.1089/cyber.2022.0044

50. Schrager SM, Mamey MR, Rhoades H, Goldbach JT. Adolescent stress experiences over time study (ASETS) protocol: design and methods of a prospective longitudinal study of sexual minority adolescents in the USA. BMJ Open. 2022 Mar;12(3):e054792.

51. Rosa GS da, Andrades GS, Caye A, Hidalgo MP, Oliveira MAB de, Pilz LK. Thirteen Reasons Why: The impact of suicide portrayal on adolescents' mental health. J Psychiatr Res. 2019 Jan;108:2–6.

52. Craig SG, Ames ME, Bondi BC, Pepler DJ. Canadian Adolescents' Mental Health and Substance Use During the COVID-19 Pandemic: Associations With COVID-19 Stressors. Can J Behav Sci. 2022 Mar 7;55(1):46–55.

53. Park BK, Calamaro C. A systematic review of social networking sites: Innovative platforms for health research targeting adolescents and young adults. J Nurs Scholarsh [Internet]. 2013;45(3):256–64. Available from: https://www.scopus.com/inward/record.uri?eid=2-s2.0-84883453371&doi=10.1111%2Fjnu.12032&partnerID=40&md5=23e56117ac2aeb8d2db3e2d4f5974631

54. Ryan GS. Online social networks for patient involvement and recruitment in clinical research. Nurse Res [Internet]. 2013;21(1):35–9. Available from: https://www.scopus.com/inward/record.uri?eid=2-s2.0-84885904750&doi=10.7748%2Fnr2013.09.21.1.35.e302&partnerID=40&md5=f47ade8b9a58738f274bcdbac6a8f59f

55. Piers R, Williams JM, Sharpe H. Can digital mental health interventions bridge the 'digital divide'for socioeconomically and digitally marginalised youth? A systematic review. Child Adolesc Ment Health. 2023;28(1):90–104.

56. Reiss F. Socioeconomic inequalities and mental health problems in children and adolescents: A systematic review. Soc Sci Med [Internet]. 2013;90:24–31. Available from: https://www.sciencedirect.com/science/article/pii/S0277953613002608

57. Metherell TE, Ghai S, McCormick EM, Ford TJ, Orben A. Digital exclusion predicts worse mental health among adolescents during COVID-19. medRxiv. 2021;2011–21.

58. Pew Research Centre. Teens, Social Media and Technology 2022 [Internet]. 2022 [cited 2023 Apr 5]. Available from: https://www.pewresearch.org/internet/2022/08/10/teens-social-media-and-technology-2022/

For peer review only

For peer review only

Figure 1: PRISMA Flow Diagram

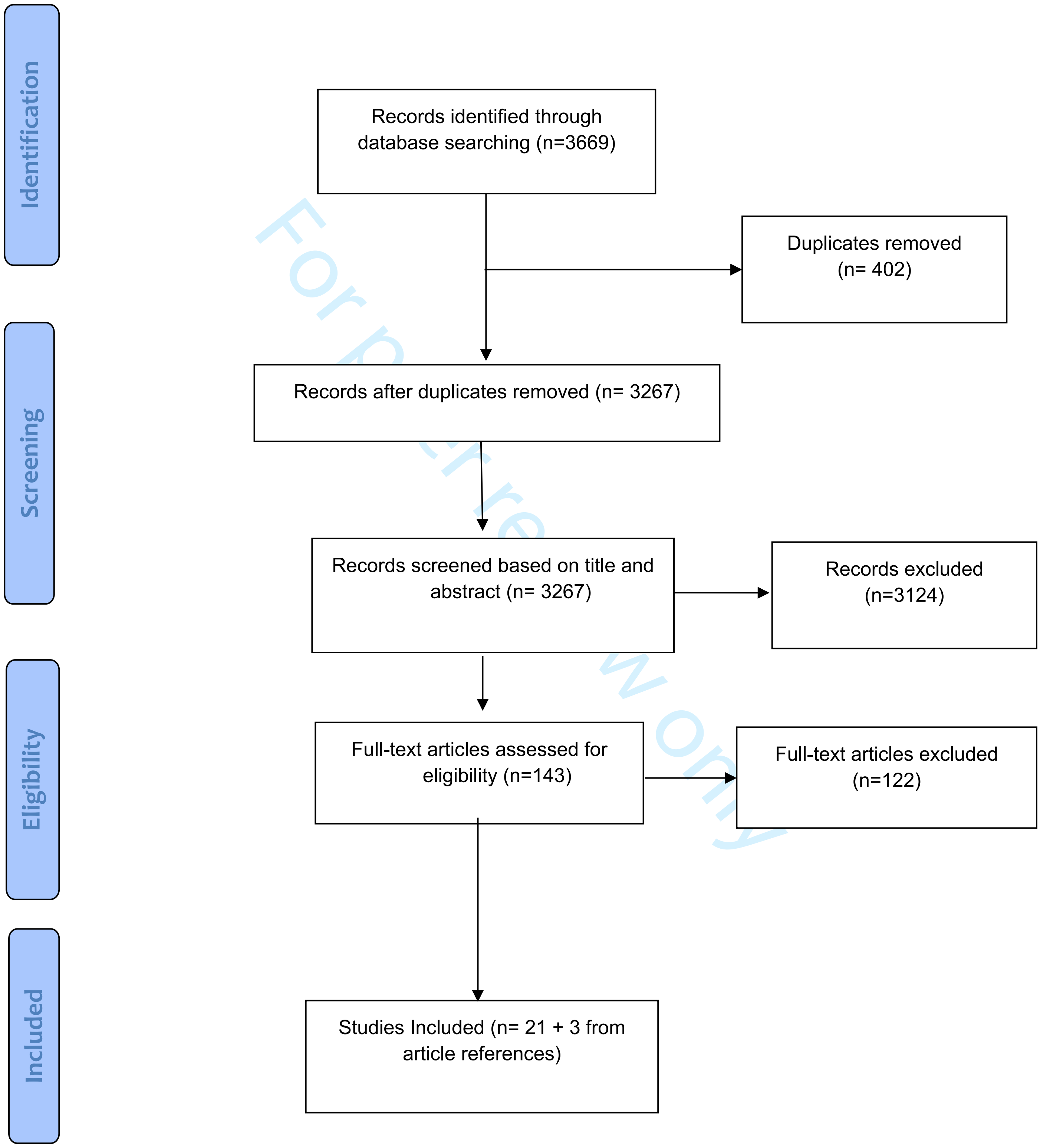

Supplementary Materials

Supplementary Material 1: Full electronic search strategy for PUBMED

Limitations:

- *2011 – Present*
- *Humans*
- *English*
- *Adolescent 13-18*

Search Terms:
(((((((((((((((((adolescent[Title/Abstract]) OR (adolescent[MeSH Major Topic])) OR (youth[Title/Abstract]) OR (teenager[Title/Abstract])) OR ("young adult"[Title/Abstract])) OR (young adult[MeSH Major Topic])) AND ("mental health"[Title/Abstract])) OR (mental health[MeSH Major Topic])) OR ("mental illness"[Title/Abstract])) OR ("mental disorder"[Title/Abstract])) OR (mental disorders[MeSH Major Topic])) AND ("social media"[Title/Abstract])) OR (social media[MeSH Major Topic])) OR ("social network*"[Title/Abstract])) OR (social networking[MeSH Major Topic])) AND (recruit*[Title/Abstract])) OR (advert*[Title/Abstract])) OR (advertising[MeSH Major Topic]))

For peer review only

Preferred Reporting Items for Systematic reviews and Meta-Analyses extension for Scoping Reviews (PRISMA-ScR) Checklist

SECTION	ITEM	PRISMA-ScR CHECKLIST ITEM	REPORTED ON PAGE #
**TITLE**			
Title	1	Identify the report as a scoping review.	1
**ABSTRACT**			
Structured summary	2	Provide a structured summary that includes (as applicable): background, objectives, eligibility criteria, sources of evidence, charting methods, results, and conclusions that relate to the review questions and objectives.	1
**INTRODUCTION**			
Rationale	3	Describe the rationale for the review in the context of what is already known. Explain why the review questions/objectives lend themselves to a scoping review approach.	2
Objectives	4	Provide an explicit statement of the questions and objectives being addressed with reference to their key elements (e.g., population or participants, concepts, and context) or other relevant key elements used to conceptualize the review questions and/or objectives.	3
**METHODS**			
Protocol and registration	5	Indicate whether a review protocol exists; state if and where it can be accessed (e.g., a Web address); and if available, provide registration information, including the registration number.	3
Eligibility criteria	6	Specify characteristics of the sources of evidence used as eligibility criteria (e.g., years considered, language, and publication status), and provide a rationale.	4
Information sources*	7	Describe all information sources in the search (e.g., databases with dates of coverage and contact with authors to identify additional sources), as well as the date the most recent search was executed.	4
Search	8	Present the full electronic search strategy for at least 1 database, including any limits used, such that it could be repeated.	Supplementary Material
Selection of sources of evidence†	9	State the process for selecting sources of evidence (i.e., screening and eligibility) included in the scoping review.	4
Data charting process‡	10	Describe the methods of charting data from the included sources of evidence (e.g., calibrated forms or forms that have been tested by the team before their use, and whether data charting was done independently or in duplicate) and any processes for obtaining and confirming data from investigators.	5
Data items	11	List and define all variables for which data were sought and any assumptions and simplifications made.	5
Critical appraisal of individual sources of evidence§	12	If done, provide a rationale for conducting a critical appraisal of included sources of evidence; describe the methods used and how this information was used in any data synthesis (if appropriate).	Click here to enter text.
Synthesis of results	13	Describe the methods of handling and summarizing the data that were charted.	5

St. Michael's
Inspired Care.
Inspiring Science.

For peer review only - http://bmjopen.bmj.com/site/about/guidelines.xhtml

SECTION	ITEM	PRISMA-ScR CHECKLIST ITEM	REPORTED ON PAGE #
**RESULTS**			
Selection of sources of evidence	14	Give numbers of sources of evidence screened, assessed for eligibility, and included in the review, with reasons for exclusions at each stage, ideally using a flow diagram.	6
Characteristics of sources of evidence	15	For each source of evidence, present characteristics for which data were charted and provide the citations.	6
Critical appraisal within sources of evidence	16	If done, present data on critical appraisal of included sources of evidence (see item 12).	Click here to enter text.
Results of individual sources of evidence	17	For each included source of evidence, present the relevant data that were charted that relate to the review questions and objectives.	7
Synthesis of results	18	Summarize and/or present the charting results as they relate to the review questions and objectives.	15
**DISCUSSION**			
Summary of evidence	19	Summarize the main results (including an overview of concepts, themes, and types of evidence available), link to the review questions and objectives, and consider the relevance to key groups.	17
Limitations	20	Discuss the limitations of the scoping review process.	18
Conclusions	21	Provide a general interpretation of the results with respect to the review questions and objectives, as well as potential implications and/or next steps.	18
**FUNDING**			
Funding	22	Describe sources of funding for the included sources of evidence, as well as sources of funding for the scoping review. Describe the role of the funders of the scoping review.	19

JBI = Joanna Briggs Institute; PRISMA-ScR = Preferred Reporting Items for Systematic reviews and Meta-Analyses extension for Scoping Reviews.

* Where *sources of evidence* (see second footnote) are compiled from, such as bibliographic databases, social media platforms, and Web sites.

† A more inclusive/heterogeneous term used to account for the different types of evidence or data sources (e.g., quantitative and/or qualitative research, expert opinion, and policy documents) that may be eligible in a scoping review as opposed to only studies. This is not to be confused with *information sources* (see first footnote).

‡ The frameworks by Arksey and O'Malley (6) and Levac and colleagues (7) and the JBI guidance (4, 5) refer to the process of data extraction in a scoping review as data charting.

§ The process of systematically examining research evidence to assess its validity, results, and relevance before using it to inform a decision. This term is used for items 12 and 19 instead of "risk of bias" (which is more applicable to systematic reviews of interventions) to include and acknowledge the various sources of evidence that may be used in a scoping review (e.g., quantitative and/or qualitative research, expert opinion, and policy document).

From: Tricco AC, Lillie E, Zarin W, O'Brien KK, Colquhoun H, Levac D, et al. PRISMA Extension for Scoping Reviews (PRISMAScR): Checklist and Explanation. Ann Intern Med. 2018;169:467–473. doi: 10.7326/M18-0850.

For peer review only - http://bmjopen.bmj.com/site/about/guidelines.xhtml

The use of social media in recruiting young people to mental health research: a scoping review

Journal:	*BMJ Open*
Manuscript ID	bmjopen-2023-075290.R1
Article Type:	Original research
Date Submitted by the Author:	31-Oct-2023
Complete List of Authors:	Smith, Megan; University of Hertfordshire<br>Grohmann, Dominique; University of Hertfordshire, Life and Medical Sciences<br>Trivedi, Daksha; University of Hertfordshire, Health and Social Work
**Primary Subject Heading**:	Mental health
Secondary Subject Heading:	Public health
Keywords:	MENTAL HEALTH, Adolescent, Patient Participation, Depression & mood disorders < PSYCHIATRY, PUBLIC HEALTH

I, the Submitting Author has the right to grant and does grant on behalf of all authors of the Work (as defined in the below author licence), an exclusive licence and/or a non-exclusive licence for contributions from authors who are: i) UK Crown employees; ii) where BMJ has agreed a CC-BY licence shall apply, and/or iii) in accordance with the terms applicable for US Federal Government officers or employees acting as part of their official duties; on a worldwide, perpetual, irrevocable, royalty-free basis to BMJ Publishing Group Ltd ("BMJ") its licensees and where the relevant Journal is co-owned by BMJ to the co-owners of the Journal, to publish the Work in this journal and any other BMJ products and to exploit all rights, as set out in our licence.

The Submitting Author accepts and understands that any supply made under these terms is made by BMJ to the Submitting Author unless you are acting as an employee on behalf of your employer or a postgraduate student of an affiliated institution which is paying any applicable article publishing charge ("APC") for Open Access articles. Where the Submitting Author wishes to make the Work available on an Open Access basis (and intends to pay the relevant APC), the terms of reuse of such Open Access shall be governed by a Creative Commons licence – details of these licences and which Creative Commons licence will apply to this Work are set out in our licence referred to above.

Other than as permitted in any relevant BMJ Author's Self Archiving Policies, I confirm this Work has not been accepted for publication elsewhere, is not being considered for publication elsewhere and does not duplicate material already published. I confirm all authors consent to publication of this Work and authorise the granting of this licence.

For peer review only

Title: The use of social media in recruiting young people to mental health research: a scoping review

Author names and affiliations: Megan V. A. Smith[1]., Dominique Grohmann[1]., Daksha Trivedi[2]

[1]Centre for Health Services and Clinical Research, University of Hertfordshire, Hertfordshire, Hatfield, United Kingdom
[2]Centre for Research in Public Health and Community Care, University of Hertfordshire, Hatfield, United Kingdom

Corresponding author: Megan Smith; m.smith25@herts.ac.uk

For peer review only

Abstract:

Objectives: This review explored the literature on the use of social media in recruiting young people, aged 13-18, to mental health research. It aimed to identify barriers and facilitators to recruitment and strategies to improve participation in future research.

Design: Scoping review.

Data sources: Articles published between January 2011 and February 2023 were searched for on PubMed, Scopus, Medline (via EBSCO host) and Cochrane Library databases.

Eligibility criteria: Studies that outlined social media as a recruitment method and recruited participants between the ages of 13 and 18 years.

Data extraction and synthesis: Data was extracted by two reviewers independently and cross-checked by a third reviewer. Data on study design, aims, participants, recruitment methods and findings related specifically to social media as a recruitment tool were collected.

Results: 24 journal articles met the inclusion criteria. Studies were predominantly surveys (n=13) conducted in the United States (n=16) recruiting via Facebook (n=16) and/or Instagram (n=14). Only nine of the included articles provided a summary of success and reviewed the efficacy of social media recruitment for young people in mental health research. Type of advertisement, the language used, time of day and the use of keywords were all found to be factors that may influence the success of recruitment through social media, however as these are based on findings from a small number of studies, such potential influences require further investigation.

Conclusion: Social media recruitment can be a successful method for recruiting young people to mental health research. Further research is needed into recruiting socioeconomically marginalised groups using this method, as well as the effectiveness of new social media platforms.

Pre-registration: Open Science Framework (OSF) Registry (https://osf.io/mak75/)

Strengths and limitations of this scoping review

- This is the first scoping review exploring social media platforms for the recruitment of young people to mental health research.
- The review used a rigorous approach to scoping and literature by following the Arksey and O'Malley (2005) five-stage framework.
- The search was conducted on studies between 2011 and 2023 and some studies that are 5-10 years old may not be as relevant now due to how quickly social media platforms change in popularity.
- Studies included were largely conducted in the US, therefore results cannot be generalised to countries where internet devices and social media may not be as easily accessible, or where nature of use is simply different.

1. INTRODUCTION

Mental health conditions are becoming significantly more common among children and young people (1). The impact of the COVID-19 pandemic on the mental health and wellbeing of children and young people has only added to the global growing concern. In 2022, over 3.5 million 6-23 year olds in England were reported to have a probable or possible mental health disorder (2) and globally, around 14% of the world's adolescents (aged 10-19 years) are recorded to live with a mental disorder (3). Consequently, there has been increasing demand for counselling services, hospital admissions for self-harm and referrals to specialist Child and Adolescent Mental Health Services (CAMHS) (4). In the UK, for example, figures have shown record numbers of referrals with the number of young people accessing mental health services reaching a new record of 708,939 in November 2022 (5). However, in the UK, and globally, many countries do not have enough staff trained for dealing with mental health and across all income groups, there are reported to be just three mental health workers per 1000 000 population (3). Adding to concern, reports do not account for the large proportion of children and young people not seeking or accessing professional for their mental health conditions (6).

There is a dearth of evidence on the effectiveness of appropriate and timely interventions to improve the mental wellbeing of children and young people (7). Consequently robust, large scale research studies are needed to bridge the gap between supply and demand of mental health treatments. Recruiting eligible and representative participants to research studies, however, can be challenging and is often the most time-consuming aspect of the study process (8,9). Participation in research is generally declining and ineffective recruitment can impact data quality, validity of research findings, and lead to premature termination of the trial (10,11). Recruitment and retention of young people for mental health research is challenging and adds another level of complexity (10,12,13). In particular, participation may be hindered by the stigma of mental illness or fear of negative consequences from self-disclosure (14,15).

Traditional recruitment strategies such as advertisements, telephone calls and mailing letters are often costly, time-consuming and ineffective in representing the target population (16,17). Alternatively, online recruitment strategies are now being adopted to improve enrolment outcomes (18). One example is the use of social media, a group of

For peer review only - http://bmjopen.bmj.com/site/about/guidelines.xhtml

mobile and internet-based applications allowing users to receive, build and share information worldwide (19). The launch of the social media platforms Myspace and Facebook in 2003 and 2004 respectively, fuelled a growth of online platforms designed to increase social interconnectivity (14). Twitter, Instagram, and Snapchat, among others, shortly followed and have become an avenue for daily consumption and dissemination of information.

Most young people are active on social networking sites. In 2022 it was reported that 62% of children aged 8-17 had profile(s) on online apps or sites, and using video-sharing platforms such as YouTube or TikTok was the most popular online activity amongst this age group (20). In many cases, those with mental health problems utilise social media platforms to seek support networks and help others (21), indicating that social media could be a successful method of engaging with young people for the purpose of mental health research.

An increasing number of studies are using online recruitment methods and the feasibility of such strategies are being explored. Facebook, in particular has been shown to be a successful recruitment tool for populations who may not respond to traditional recruitment strategies, such as adolescents (22,23). More specifically, the use of paid Facebook advertising, search tool and creation and use of a Facebook Page prove successful in recruiting adolescents to health research (22). Previous reviews that have explored social media platforms in addition to Facebook, found them to be useful for recruitment to mental health research (14,24) however these reviews did not target young people and therefore cannot be generalised to this population.

To date, and to our knowledge, a review has not explored social media platforms for the recruitment of young people to mental health research. The aim of this scoping review is to explore the literature on the use of social media in recruiting young people to mental health research studies and to identify barriers and facilitators to recruitment and strategies to improve participation.

2.0. METHODS

Scoping reviews aim to map all of the relevant literature in a specific area of interest and consequently help to identify any gaps in existing research (25). This approach was adopted as existing knowledge of the literature suggested a lack of previous work on how social media is used to recruit young people to mental health research.

This review utilizes the Arksey and O'Malley framework (25) for scoping reviews which involves five stages; (1) identifying the research question; (2) identifying the relevant studies; (3) study selection; (4) charting the data; (5) collating, summarizing and reporting the results. This scoping review was also conducted in line with the Preferred Reporting Items for Systematic Reviews and Meta Analysis Extension for Scoping Reviews (PRISMA-ScR) (26,27). The methods of this scoping review were pre-registered on the Open Science Framework (OSF) Registry (https://osf.io/mak75/).

2.1. Stage 1: Identifying the research questions

For peer review only

Four research questions directed this scoping review to address current gaps in the literature:

1) What social media platforms are described in the literature for recruiting young people for mental health research?
2) How are social media platforms used to recruit young people for mental health research?
3) What are the barriers and facilitators to recruitment of young people to mental health research using social media?
4) What are the strategies for improving recruitment and participation of young people to mental health research using social media?

2.2. Stage 2: Identifying relevant studies

On the 17th February 2022, searches of the following four databases were carried out: PubMed, Scopus, Medline (via EBSCO host) and Cochrane Library. The search was repeated on 3rd February 2023 to identify relevant papers published since February 2022. Medical subject headings (MeSH) were searched using Boolean operators *"OR/AND"*. The search terms were: (adolescent OR teenager OR youth OR "young adult") AND ("mental health" OR "mental illness*" OR "mental disorder") AND ("social media" OR "social network*") AND (recruit* OR advert*). The search format used in the databases was modified to meet their requirements. Further details regarding the search terms used are provided in Table 1. The final search strategy for databases can be found in supplementary file 1.

Table 1: Search terms

Search term 1	Search term 2	Search term 3	Search term 4
Search operator	*AND*	*AND*	*AND*
Adolescent	"Mental health"	"Social media"	Recruit*
Adolescent	Mental Health	Social media	Advert*
Teenager	"Mental illness*"	"Social network*"	Advertising
Youth	Mental disorders	Social Networking	
"Young adult"	Mental disorder		
Young adult			

MeSH terms [MeSH major topic]

As past reviews in this area have often focused on the use of Facebook (e.g. (18,22)) this review aimed to capture publications exploring all social media platforms developed since. As Instagram saw significant growth in December 2010, articles published prior to 1st January 2011 were not included in the database searches, allowing time for any use of Instagram for recruitment to be reported. Therefore, the review years spanned from January 2011 to February 2023.

2.3. Stage 3: Study selection

For peer review only - http://bmjopen.bmj.com/site/about/guidelines.xhtml

Studies were included if they (a) outlined the use of social media (e.g., Twitter, Instagram, Facebook, Snapchat and TikTok) as a method to recruit young people aged 13 to 18 and/or those with a mean age of 13-18 (b) addressed a mental ill-health (e.g., depression, anxiety or eating disorder) (c) were published between January 2011 and February 2023. Studies were excluded from this review if they met any of the following criteria:

1. Did not refer to the use of social media for recruitment of young people aged 13 to 18 in the methodology
2. Were not written in the English language (due to the time and cost involved in translating them to English)
3. Did not address a mental ill-health

Retrieved records from the database searches were extracted and imported into Rayyan, a free web tool created to facilitate literature reviews (28). One reviewer (MS) scanned all imported records and removed duplicates. Titles and abstracts were screened to define the eligibility of each article by two researchers (MS, DG) independently following the aforementioned pre-defined selection criteria. Publications with a title or abstract not meeting the eligibility criteria were excluded. Following the initial screening, full-text articles were reviewed by both researchers independently to make a final decision of inclusion. Any discrepancies regarding the inclusion of articles were resolved by discussion and mutual agreement with the third reviewer (DT). The full text of relevant papers was retrieved for further analysis by two reviewers (MS, DG) and was either included or excluded for review based on the eligibility criteria presented below. Primary studies and/or relevant systematic reviews that met the inclusion criteria were included. However, due to the broad age range of two relevant systematic reviews, included articles were individually screened for eligibility. From this, one article met inclusion criteria for the current scoping review and was consequently included in analysis.

The reference lists of all included publications were also examined to ensure all relevant and eligible resources had been identified.

2.4. Stage 4: Charting the data

A data extraction form was developed in Excel by one of the reviewers and agreed by all to determine which variables to extract. Relevant articles were charted using the following column headings:

1. Author(s), year of publication
2. Study design
3. Study aim(s)
4. Participants
5. Recruitment method(s)
6. Social media platforms
7. Recruitment related findings

The two reviewers independently charted the data, discussed the results, and continuously updated the data-charting form in an iterative process.

For peer review only

2.5. Stage 5: Collating, summarizing and reporting the results

All extracted data from the included articles were summarised and tabulated by a member of the research team. In line with Arksey and O'Malley (25), the narrative account is presented in two ways. Firstly, the nature and distribution of the studies included, e.g., study design, country, settings, and participants group were reported. Secondly, the literature was organised according to the following themes which were drawn from the research questions: methods of social media recruitment to recruit young people to mental health research, barriers and facilitators to recruitment of young people to mental health research using social media, how to improve recruitment and participation of young people to mental health research using social media.

2.6. Patient and Public Involvement

There was no patient and public involvement due to the nature of this research.

2. RESULTS

2.1. Included studies

The searches in 2022 and 2023 are presented together as the same search methodology was adopted. Initial searching of the electronic databases provided 3,669 records. Four hundred and two duplicates were removed leaving 3,267 records to screen for relevance based on title and abstract. Based on title and abstract screening, 3,124 were excluded. One hundred and forty-three full-text articles were assessed by the researchers for inclusion. One hundred and twenty-two were excluded. The majority of articles were excluded due to not addressing mental-ill health or not including the correct sample i.e., young people aged 13-18. Other reasons for exclusion included incorrect study design or recruitment technique. A total of 21 studies met all inclusion criteria and were subsequently included within this scoping review. When screening the references of the 21 included articles, 2 articles (29,30) were found to fit the inclusion criteria and were therefore also included within the review. As mentioned previously, one paper (31) was included following the screening of two potentially eligible systematic reviews. An overview of the study selection process is provided in Figure 1.

2.2. Study characteristics

Characteristics of the included articles are summarised in supplementary material file 2. Study designs included 13 surveys (29–41), three pilot randomised controlled trials (42–44), four feasibility studies (45–48), two randomised controlled trials (49,50) and one mixed methods exploratory study (51). One paper (52) presents the protocol of a longitudinal study utilising a subset of Goldbach et al's (38) sample. Schrager et al., (52) provide detailed information of the recruitment methods used by Goldbach et al (38). For the purpose of this review, they are presented as separate studies.

For peer review only

Of the included articles, 16 were conducted in the United States (29,33,37–48,50,52), three in Canada (31,34,35), one in Australia (51), one in Sweden (49) and one in Brazil (53). One of the included articles recruited participants from both the United States and Brazil (30), and one recruited participants from "major English-speaking countries" including Australia, United States, the United Kingdom and New Zealand (32).

2.3. Participants

Some articles grouped their entire sample by age (33,34,37,41), therefore only the data from the groups that were eligible for this review were considered (13–18-year-olds). Four studies recruited youth with a mental health condition (32,45,47,49) and two recruited athletes (39,40). Nine studies recruited adolescents from specific demographic backgrounds e.g., sexual and gender minorities (29,34,38,44,48,52), America Indian and Alaska Native (AI/AN) youth (50) and heterosexually active Black youth (42). Four studies recruited youth based on social media activity i.e. posting online about being sad or depressed (33,43), females engaging in pro-eating disorder posts (37), individuals who had liked online social media pages for the television series "13 Reasons Why" (30,36). One study recruited youth who had been affected by violence (31) and one recruited youth seeking support for issues related to family discord and associated impacts on emotional well-being (51). The remaining three studies recruited youth with no particular characteristics noted (41,46,54).

2.4. Methods of social media recruitment

Among all retrieved articles, 20 described at least one specific type of social media as a method of recruitment (see supplementary file 2 for distribution). Facebook was used most commonly by 16 of the 24 included articles (30–33,35–41,45,48,50–52), 14 used Instagram (29,33,35,37,38,41,44–48,50–52) and 5 used Twitter (32,33,37,39,40). Other, less commonly used social media platforms were Reddit (33,37,45), Tumblr (30,33,43), Snapchat (45,51), TikTok (45) and YouTube (38,45,52). Three articles, although discussing the use of social media for recruitment, did not specify the platforms used (34,42,49).

Of the articles which specified the platforms used, 12 of these used more than one social media platform to advertise (29,32,33,37–41,45,48,50,52). Half of the articles (n=12) used advertisements tailored to reach their target population by setting restrictions, for example keywords, age, interests or location (29,30,32,33,37,38,41,45,46,48,50,52). Three articles specified using Facebook Business Manager to manage Instagram and/or Facebook advertisements (45,46,50).

Some articles used a combination of online and offline advertisement methods. Brawner et al., (42) for example, initially aimed to recruit through community-based mental health providers, high schools, community partners (e.g., recreation centres) and provider referrals, however, this was not successful so introduced social media study promotion. Lattie et al., (47) advertised via Instagram as well as offline e.g., through schools, community settings, fliers, university databases and at conferences. Morgan et al., (32) also used a combination of paid advertisements on Facebook and Google as well as hard copy advertisements in youth mental health clinics and support groups. Amon et al., (51) recruited specifically through the Kids Helpline (KHL) website, referrals from KHL counsellors, and schools, as well as through social media. Goldbach et al., (38) and Schrager

For peer review only

et al. (52) used social media advertising along with respondent-driven sampling, where participants could earn gift cards through referring eligible participants. Mechler et al., (49) also recruited through contacts with schools, youth associations, social workers and healthcare providers.

2.5. Barriers to social media recruitment

Few articles discussed barriers to social media recruitment. One potential barrier highlighted was that this method of recruitment may be more biased to individuals with a higher socioeconomic status who have access to internet facilities and therefore likely to be active on social media (31,35,39,40) potentially leading to an unrepresentative sample. Cost may be seen as a potential barrier, and several studies revealed their total cost of using social media advertising: $1,351 (31); $1,536 (48); $1,591 (46); over $5,000 (50). One study highlighted the potential complexity of using social media advertising, stating that using Facebook Business Manager to monitor their advert statistics could be complicated (46).

2.6. Facilitators of social media recruitment

Six of the 24 included articles discussed facilitators of social media recruitment in detail. Regarding most effective type of social media platform for recruitment, Kasson et al., (45) found Snapchat to be the most successful, with Instagram as the second most successful in a comparison of five social media sites. Fitzsimmons-Craft et al., (37) also found Instagram to be highly effective at recruiting their adolescent age group (15–17-year-olds) with 76% of the sample recruited through this platform. One study Kutok et al., (46) compared combinations of Instagram advertisements and found that those with the campaign strategy "Traffic" (targeting users that often click on links within an ad) and the advertisement placement "Feed" (on Instagram's regular feed of posts, as opposed to a "Story" which disappears after 24 hours) were the most effective combination in terms of recruitment success and cost-effectiveness. Regarding wording of social media advertisements, two studies found that those with a more positive tone or those that suggested helping the researchers received more interest (46,50). Chu and Snider (31) investigated Facebook advertising throughout the week and found the number of clicks and impressions were much higher on weekends than weekdays. One study by Kelleher et al., (43) successfully recruited youth through Tumblr who had posted about depression, through searching #depress to identify potentially suitable individuals. Several studies made use of "labels" or "keywords" in order to target a certain population (37,45,48), however the effectiveness of this strategy in aiding recruitment is difficult to determine as there were no comparator advertisements.

2.7. How to improve recruitment and participation of young people to mental health research using social media

Few articles addressed methods to improve recruitment of this population group using social media. Several studies found that advertising through Instagram was beneficial in their recruitment, which suggests that it may be a suitable platform in attracting young people to mental health research (37,45). Snapchat was also found to be highly effective at recruiting (45). Both of these social media platforms are heavily image based which may

For peer review only

have led to the increase in interest and is a factor to consider in future research. Kutok et al., (46) noted that social media is an ever-changing environment and therefore highlighted the importance of being flexible. For example, Instagram and Snapchat may be effective currently, however in several years' time other platforms may be more popular and therefore researchers should be open to trying a range of platforms. Several studies found that adverts with a more positive tone received more interest (46,50), which suggests that the language used when advertising the study should be an important consideration for future studies.

3. DISCUSSION

3.1. Main findings

The aim of this scoping review was to explore the literature on the use of social media in recruiting young people to mental health research and to identify barriers and facilitators to recruitment and strategies for improving recruitment. From the 3,308 articles identified through the four databases, 24 studies met the inclusion criteria.

The included studies investigated a range of populations recruited successfully, suggesting that social media may be a suitable method for engaging with and recruiting specific populations. This is consistent with previous reviews investigating social media as a recruitment method for health research (55,56). However, further research is required to determine the suitability of digital delivery to socioeconomically and digitally marginalised youth to mental health research (57). This is even more crucial considering socioeconomically marginalised, and consequently digitally excluded, youth are more likely to develop mental health problems than more socioeconomically advantaged peers (57–59).

Regarding type of social media platform, the majority of studies used the platform Facebook, consistent with previous reviews focusing on the general population (22,23). Facebook use in 2014-2015 amongst teens was at 71%, which has reduced considerably to 32% in 2021 (60). This decrease in popularity could imply that other social media platforms may be preferred when targeting this population and highlights the importance of keeping up to date with the most popular platforms with this age group at the time of recruitment. Instagram was also a popular platform of choice. Kasson and colleagues (45) found Snapchat and Instagram to be the most effective social media sites regarding percentage of the sample recruited. They suggested that the nature of these sites being heavily image based may attract this population compared with other networking sites. However, few of the studies compared effectiveness across a range of platforms, therefore these findings should be interpreted with caution and comparison of sites is something that should be investigated further in the future.

Regarding barriers to using social media as a recruitment technique, one of the main concerns is the potential cost of advertising, with included studies ranging from $1,351 to over $5,000. Affordability will largely depend on decisions made by the research team and the funding that has been allocated, however this highlights a need for future studies to investigate cost-effectiveness of social media compared to other forms of recruitment to determine which is more appropriate, particularly if teams have a limited budget. Another

For peer review only - http://bmjopen.bmj.com/site/about/guidelines.xhtml

potential barrier to using social media for recruitment is the potential complexity of advertising sites, however this is likely to vary depending on the research team's knowledge and experience in this area. An additional barrier and potential consideration are the caveats of minimum age requirements to sign up to social networking sites, as well as specific considerations around consent and confidentiality. This must not be overlooked if social media are to be used in adolescent research (22).

3.2. Strengths and limitations

To our knowledge, this is the first scoping review exploring all social media platforms for the recruitment of young people to mental health research. Furthermore, it followed the rigorous Arksey and O'Malley (2005) five-stage framework which allowed for transparency. To appreciate the findings of this review the following limitations should be acknowledged. Firstly, not all published articles will necessarily discuss recruitment in the title or abstract and therefore some articles may have been overlooked by the search. It is important to also note that the search was conducted from 2011 to 2023 and due to the ever-changing nature of popularity of social media sites, some of the studies that were conducted several years ago may not be as relevant now. Another limitation is that all included studies were from Western countries such as Canada and the United States, therefore the results cannot be generalised to countries in which social media use is restricted or different from these countries. Some of the social media platforms investigated here are banned in countries including Iran, China and Uganda, therefore results relating to certain platforms may not be applicable. Finally, only a few of the papers included reported data on the specifics of using social media as a recruitment method, for example the days that are best to advertise, tone of advert and any keywords used, therefore these findings should be interpreted with caution.

3.3. Future suggestions

Although the findings and reflections from these papers may be of help for other researchers who are looking to recruit using social media, many of those included did not evaluate the effectiveness of social media as a recruitment technique. Therefore, we suggest that future studies provide more detail and report on the effectiveness of various strategies adopted such as the platform, type of advertisement and language used, as well as the number of participants recruited through social media compared to more traditional recruitment methods if these are also adopted. As this method of recruitment increases in popularity, a systematic review could be conducted to include a more comprehensive search to ensure those publications not mentioning recruitment methods in their title and abstract are not disregarded from the search. Additionally, it would be beneficial for researchers to investigate the cost-effectiveness of social media compared to other types of recruitment to ascertain whether it is a cost-effective method. Such findings may then contribute towards developing a guidance document on this topic that can be shared amongst researchers to increase knowledge and awareness when considering this recruitment method for their research. As mentioned, the popularity of social media platforms can change over time and consequently so can the effectiveness of their use in recruitment. Therefore, future research could look to explore recruitment trends across different platforms, countries and mental health conditions longitudinally.

For peer review only

4. CONCLUSIONS

Recruiting young people to mental health research can be challenging and increasingly, online recruitment methods are being used to increase enrolment and accessibility to participants. This review concludes that social media can be a successful method for recruiting young people to mental health research in terms of reaching the target number of participants, recruiting specific populations and in some cases be more successful compared with traditional methods of recruitment. Further research is needed into recruiting socioeconomically marginalised groups using this method, as well as the effectiveness of new social media platforms. As technology continues to advance, so must recruitment methods.

Figures Figure 1: PRISMA Flow Diagram

Acknowledgments The authors would like to thank Lauren Denyer for her assistance in developing the protocol for this scoping review.

Author contributions MS and DG contributed to the conception and design of this review. MS conducted the search and selection process. MS and DG extracted all data. DT provided advice and guidance on the analysis and interpretation of results. MS and DG produced the first draft of the manuscript. All authors contributed to writing and approved the final draft of the manuscript.

Competing interests None declared.

Funding This work was supported by the Health Technology Assessment funding stream of the National Institutes of Health Research (NIHRA) (Reference Number: 17/78/10).

Data Sharing Data sharing not applicable as no datasets generated and/or analysed for this study.

Ethical Approval Statement This study does not involve human participants.

REFERENCES

1. Hossain MM, Nesa F, Das J, Aggad R, Tasnim S, Bairwa M, et al. Global burden of

For peer review only

mental health problems among children and adolescents during COVID-19 pandemic: An umbrella review. Psychiatry Res. 2022;114814.

2. Young Minds. Impact Report [Internet]. 2023. Available from: https://www.youngminds.org.uk/about-us/reports-and-impact/impact-report-2022/highlight-tour/

3. Organization WH. World mental health report: transforming mental health for all. 2022;

4. Pitchforth J, Fahy K, Ford T, Wolpert M, Viner RM, Hargreaves DS. Mental health and well-being trends among children and young people in the UK, 1995-2014: analysis of repeated cross-sectional national health surveys. Psychol Med. 2019 Jun;49(8):1275–85.

5. NHS Digital. Mental Health Services Monthly Statistics [Internet]. 2023. Available from: https://digital.nhs.uk/data-and-information/data-collections-and-data-sets/data-sets/mental-health-services-data-set/statistics-and-reports

6. Radez J, Reardon T, Creswell C, Lawrence PJ, Evdoka-Burton G, Waite P. Why do children and adolescents (not) seek and access professional help for their mental health problems? A systematic review of quantitative and qualitative studies. Eur Child Adolesc Psychiatry [Internet]. 2021;30(2):183–211. Available from: https://doi.org/10.1007/s00787-019-01469-4

7. Schley C, Pace N, Mann R, McKenzie C, McRoberts A, Parker A. The headspace Brief Interventions Clinic: Increasing timely access to effective treatments for young people with early signs of mental health problems. Early Interv Psychiatry. 2019;13(5):1073–82.

8. Orri M, Lipset CH, Jacobs BP, Costello AJ, Cummings SR. Web-based trial to evaluate the efficacy and safety of tolterodine ER 4 mg in participants with overactive bladder: REMOTE trial. Contemp Clin Trials. 2014;38(2):190–7.

9. Moseson H, Kumar S, Juusola JL. Comparison of study samples recruited with virtual versus traditional recruitment methods. Contemp Clin trials Commun. 2020;19:100590.

10. Hoffmann SH, Folker AP, Buskbjerg M, Folker MP, Jezek AH, Svarta DL, et al. Potential of Online Recruitment Among 15-25-Year Olds: Feasibility Randomized Controlled Trial. JMIR Form Res. 2022;6(5):e35874.

11. Bull J, Uhlenbrauck G, Mahon E, Furlong P, Roberts J. Barriers to Trial Recruitment and Possible Solutions. Appl Clin Trials. 2016;25(2).

12. Moreno MA, Waite A, Pumper M, Colburn T, Holm M, Mendoza J. Recruiting Adolescent Research Participants: In-Person Compared to Social Media Approaches. Cyberpsychol Behav Soc Netw. 2017 Jan;20(1):64–7.

13. Liu Y, Pencheon E, Hunter RM, Moncrieff J, Freemantle N. Recruitment and retention strategies in mental health trials–a systematic review. PLoS One. 2018;13(8):e0203127.

14. Sanchez C, Grzenda A, Varias A, Widge AS, Carpenter LL, McDonald WM, et al. Social media recruitment for mental health research: a systematic review. Compr Psychiatry. 2020;103:152197.

For peer review only

15. Woodall A, Morgan C, Sloan C, Howard L. Barriers to participation in mental health research: are there specific gender, ethnicity and age related barriers? BMC Psychiatry. 2010;10(1):1–10.

16. Surdam J, Daly B, Fulton S, Margevisius S, Schluchter M, Flocke S, et al. Recruitment strategies for nurse enrollment in an online study. Nurs Res. 2020;69(1):69.

17. Bethel C, Rainbow JG, Dudding KM. Recruiting nurses via social media for survey studies. Nurs Res. 2021;70(3):231–5.

18. Thornton L, Batterham PJ, Fassnacht DB, Kay-Lambkin F, Calear AL, Hunt S. Recruiting for health, medical or psychosocial research using Facebook: Systematic review. Internet Interv. 2016;4:72–81.

19. Cataldo I, Lepri B, Neoh MJY, Esposito G. Social media usage and development of psychiatric disorders in childhood and adolescence: a review. Front psychiatry. 2021;11:1332.

20. Ofcom. Children and parents: media use and attitudes report 2022 [Internet]. 2022. Available from: https://www.ofcom.org.uk/__data/assets/pdf_file/0024/234609/childrens-media-use-and-attitudes-report-2022.pdf

21. Naslund JA, Aschbrenner KA, Marsch LA, Bartels SJ. The future of mental health care: peer-to-peer support and social media. 2045; Available from: https://doi.org/10.1017/S2045796015001067

22. Amon KL, Campbell AJ, Hawke C, Steinbeck K. Facebook as a recruitment tool for adolescent health research: a systematic review. Acad Pediatr. 2014;14(5):439-447.e4.

23. Amon KL, Paxton K, Klineberg E, Riley L, Hawke C, Steinbeck K. Insights into Facebook Pages: an early adolescent health research study page targeted at parents. Int J Adolesc Med Health. 2016 Feb;28(1):69–77.

24. Bour C, Ahne A, Schmitz S, Perchoux C, Dessenne C, Fagherazzi G. The use of social media for health research purposes: scoping review. J Med Internet Res. 2021;23(5):e25736.

25. Arksey H, O'Malley L. Scoping studies: towards a methodological framework. https://doi.org/101080/1364557032000119616 [Internet]. 2007 Feb [cited 2022 Sep 22];8(1):19–32. Available from: https://www.tandfonline.com/doi/abs/10.1080/1364557032000119616

26. Tricco AC, Lillie E, Zarin W, O'Brien KK, Colquhoun H, Levac D, et al. PRISMA extension for scoping reviews (PRISMA-ScR): checklist and explanation. Ann Intern Med. 2018;169(7):467–73.

27. Peters MDJ, Marnie C, Tricco AC, Pollock D, Munn Z, Alexander L, et al. Updated methodological guidance for the conduct of scoping reviews. JBI Evid Synth [Internet]. 2020;18(10). Available from: https://journals.lww.com/jbisrir/Fulltext/2020/10000/Updated_methodological_guidance_for_the_conduct_of.4.aspx

28. Ouzzani M, Hammady H, Fedorowicz Z, Elmagarmid A. Rayyan---a web and mobile app for systematic reviews. Syst Rev [Internet]. 2016;5(1):210. Available from:

For peer review only

http://dx.doi.org/10.1186/s13643-016-0384-4

29. Smith DM, Wang SB, Carter ML, Fox KR, Hooley JM. Longitudinal predictors of self-injurious thoughts and behaviors in sexual and gender minority adolescents. J Abnorm Psychol. 2020;129(1):114.

30. Zimerman A, Caye A, Salum GA, Passos IC, Kieling C. Revisiting the Werther effect in the 21st century: bullying and suicidality among adolescents who watched 13 Reasons Why. J Am Acad Child Adolesc Psychiatry. 2018;57(8):610–3.

31. Chu JL, Snider CE. Use of a social networking web site for recruiting Canadian youth for medical research. J Adolesc Heal Off Publ Soc Adolesc Med. 2013 Jun;52(6):792–4.

32. Morgan AJ, Ross AM, Yap MBH, Reavley NJ, Parker A, Simmons MB, et al. What works for mental health problems in youth? Survey of real-world experiences of treatments and side effects. Early Interv Psychiatry. 2021;15(6):1502–12.

33. Szlyk H, Deng J, Xu C, Krauss MJ, Cavazos-Rehg PA. Leveraging social media to explore the barriers to treatment among individuals with depressive symptoms. Depress Anxiety [Internet]. 2020;37(5):458 – 465. Available from: https://www.scopus.com/inward/record.uri?eid=2-s2.0-85077903892&doi=10.1002%2Fda.22990&partnerID=40&md5=475bbd981c9ef52a9c2a2fb19bef010d

34. Veale JF, Watson RJ, Peter T, Saewyc EM. Mental Health Disparities Among Canadian Transgender Youth. J Adolesc Heal Off Publ Soc Adolesc Med. 2017 Jan;60(1):44–9.

35. Craig SG, Ames ME, Bondi BC, Pepler DJ. Canadian adolescents' mental health and substance use during the COVID-19 pandemic: Associations with COVID-19 stressors. Vol. 55, Canadian Journal of Behavioural Science / Revue canadienne des sciences du comportement. Craig, Stephanie G.: Department of Psychology, York University, 4700 Keele Street, Toronto, ON, Canada, M3J 1P3, sgcraig@yorku.ca: Educational Publishing Foundation; 2023. p. 46–55.

36. da Rosa GS, Andrades GS, Caye A, Hidalgo MP, de Oliveira MAB, Pilz LK. Thirteen Reasons Why: The impact of suicide portrayal on adolescents' mental health. J Psychiatr Res. 2019;108:2–6.

37. Fitzsimmons-Craft EE, Krauss MJ, Costello SJ, Floyd GM, Wilfley DE, Cavazos-Rehg PA. Adolescents and young adults engaged with pro-eating disorder social media: eating disorder and comorbid psychopathology, health care utilization, treatment barriers, and opinions on harnessing technology for treatment. Eat Weight Disord. 2020 Dec;25(6):1681–92.

38. Goldbach JT, Parra LA, O'Brien RP, Rhoades H, Schrager SM. Explaining behavioral health differences in urban and rural sexual minority adolescents: A longitudinal investigation of minority stress in a diverse national sample of sexual minority adolescents: A longitudinal investigation of minority stress in a div. J Rural Heal Off J Am Rural Heal Assoc Natl Rural Heal Care Assoc. 2023 Jan;39(1):262–71.

39. McGuine TA, Biese KM, Petrovska L, Hetzel SJ, Reardon CL, Kliethermes S, et al. Changes in the Health of Adolescent Athletes: A Comparison of Health Measures Collected before and during the COVID-19 Pandemic. J Athl Train. 2021;56(8):836–44.

For peer review only

40. McGuine TA, Biese KM, Petrovska L, Hetzel SJ, Reardon C, Kliethermes S, et al. Mental Health, Physical Activity, and Quality of Life of US Adolescent Athletes During COVID-19-Related School Closures and Sport Cancellations: A Study of 13 000 Athletes. J Athl Train. 2021 Jan;56(1):11–9.

41. Mitchell KJ, Ybarra ML, Banyard V, Goodman KL, Jones LM. Impact of the COVID-19 Pandemic on Perceptions of Health and Well-Being Among Sexual and Gender Minority Adolescents and Emerging Adults. LGBT Heal. 2022 Jan;9(1):34–42.

42. Brawner BM, Jemmott LS, Hanlon AL, Lozano AJ, Abboud S, Ahmed C, et al. Results from Project GOLD: A pilot randomized controlled trial of a psychoeducational HIV/STI prevention intervention for black youth. AIDS Care. 2021 Jun;33(6):767–85.

43. Kelleher E, Moreno M, Wilt MP. Recruitment of participants and delivery of online mental health resources for depressed individuals using tumblr: Pilot randomized control trial. JMIR Res Protoc [Internet]. 2018;7(4). Available from: https://www.scopus.com/inward/record.uri?eid=2-s2.0-85081410138&doi=10.2196%2Fresprot.9421&partnerID=40&md5=7775ffd7c27cd89f6ef4c9f59575d5a0

44. Bauermeister J, Choi SK, Bruehlman-Senecal E, Golinkoff J, Taboada A, Lavra J, et al. An Identity-Affirming Web Application to Help Sexual and Gender Minority Youth Cope With Minority Stress: Pilot Randomized Controlled Trial. J Med Internet Res [Internet]. 2022 Aug 1 [cited 2023 Mar 29];24(8). Available from: https://pubmed.ncbi.nlm.nih.gov/35916700/

45. Kasson E, Vázquez MM, Doroshenko C, Fitzsimmons-Craft EE, Wilfley DE, Taylor CB, et al. Exploring Social Media Recruitment Strategies and Preliminary Acceptability of an mHealth Tool for Teens with Eating Disorders. Int J Environ Res Public Health. 2021 Jul;18(15).

46. Kutok ER, Doria N, Dunsiger S, Patena J V, Nugent NR, Riese A, et al. Feasibility and Cost of Using Instagram to Recruit Adolescents to a Remote Intervention. J Adolesc Heal Off Publ Soc Adolesc Med. 2021 Nov;69(5):838–46.

47. Lattie EG, Ho J, Sargent E, Tomasino KN, Smith JD, Brown CH, et al. Teens engaged in collaborative health: The feasibility and acceptability of an online skill-building intervention for adolescents at risk for depression. Internet Interv [Internet]. 2017;8:15 – 26. Available from: https://www.scopus.com/inward/record.uri?eid=2-s2.0-85014559187&doi=10.1016%2Fj.invent.2017.02.003&partnerID=40&md5=ca5264fed2384526cf9e3bf57f35fe90

48. Salk RH, Thoma BC, Choukas-Bradley S. The Gender Minority Youth Study: Overview of Methods and Social Media Recruitment of a Nationwide Sample of U.S. Cisgender and Transgender Adolescents. Arch Sex Behav. 2020 Oct;49(7):2601–10.

49. Mechler J, Lindqvist K, Carlbring P, Topooco N, Falkenström F, Lilliengren P, et al. Therapist-guided internet-based psychodynamic therapy versus cognitive behavioural therapy for adolescent depression in Sweden: a randomised, clinical, non-inferiority trial. Lancet Digit Heal. 2022 Aug 1;4(8):e594–603.

50. Stephens D, Peterson R, Singer M, Johnson J, Rushing SC, Kelley A. Recruiting and Engaging American Indian and Alaska Native Teens and Young Adults in a SMS Help-

Seeking Intervention: Lessons Learned from the BRAVE Study. Int J Environ Res Public Health. 2020 Dec;17(24).

51. Amon K, Ridout B, Forsyth R, Campbell A. Online Group Counseling for Young People Through a Customized Social Networking Platform: Phase 2 of Kids Helpline Circles. https://home.liebertpub.com/cyber [Internet]. 2022 Sep 14 [cited 2023 Mar 29];25(9):580–8. Available from: https://www.liebertpub.com/doi/10.1089/cyber.2022.0044

52. Schrager SM, Mamey MR, Rhoades H, Goldbach JT. Adolescent stress experiences over time study (ASETS) protocol: design and methods of a prospective longitudinal study of sexual minority adolescents in the USA. BMJ Open. 2022 Mar;12(3):e054792.

53. Rosa GS da, Andrades GS, Caye A, Hidalgo MP, Oliveira MAB de, Pilz LK. Thirteen Reasons Why: The impact of suicide portrayal on adolescents' mental health. J Psychiatr Res. 2019 Jan;108:2–6.

54. Craig SG, Ames ME, Bondi BC, Pepler DJ. Canadian Adolescents' Mental Health and Substance Use During the COVID-19 Pandemic: Associations With COVID-19 Stressors. Can J Behav Sci. 2022 Mar 7;55(1):46–55.

55. Park BK, Calamaro C. A systematic review of social networking sites: Innovative platforms for health research targeting adolescents and young adults. J Nurs Scholarsh [Internet]. 2013;45(3):256–64. Available from: https://www.scopus.com/inward/record.uri?eid=2-s2.0-84883453371&doi=10.1111%2Fjnu.12032&partnerID=40&md5=23e56117ac2aeb8d2db3e2d4f5974631

56. Ryan GS. Online social networks for patient involvement and recruitment in clinical research. Nurse Res [Internet]. 2013;21(1):35–9. Available from: https://www.scopus.com/inward/record.uri?eid=2-s2.0-84885904750&doi=10.7748%2Fnr2013.09.21.1.35.e302&partnerID=40&md5=f47ade8b9a58738f274bcdbac6a8f59f

57. Piers R, Williams JM, Sharpe H. Can digital mental health interventions bridge the 'digital divide'for socioeconomically and digitally marginalised youth? A systematic review. Child Adolesc Ment Health. 2023;28(1):90–104.

58. Reiss F. Socioeconomic inequalities and mental health problems in children and adolescents: A systematic review. Soc Sci Med [Internet]. 2013;90:24–31. Available from: https://www.sciencedirect.com/science/article/pii/S0277953613002608

59. Metherell TE, Ghai S, McCormick EM, Ford TJ, Orben A. Digital exclusion predicts worse mental health among adolescents during COVID-19. medRxiv. 2021;2011–21.

60. Pew Research Centre. Teens, Social Media and Technology 2022 [Internet]. 2022 [cited 2023 Apr 5]. Available from: https://www.pewresearch.org/internet/2022/08/10/teens-social-media-and-technology-2022/

For peer review only

For peer review only

Figure 1: PRISMA Flow Diagram

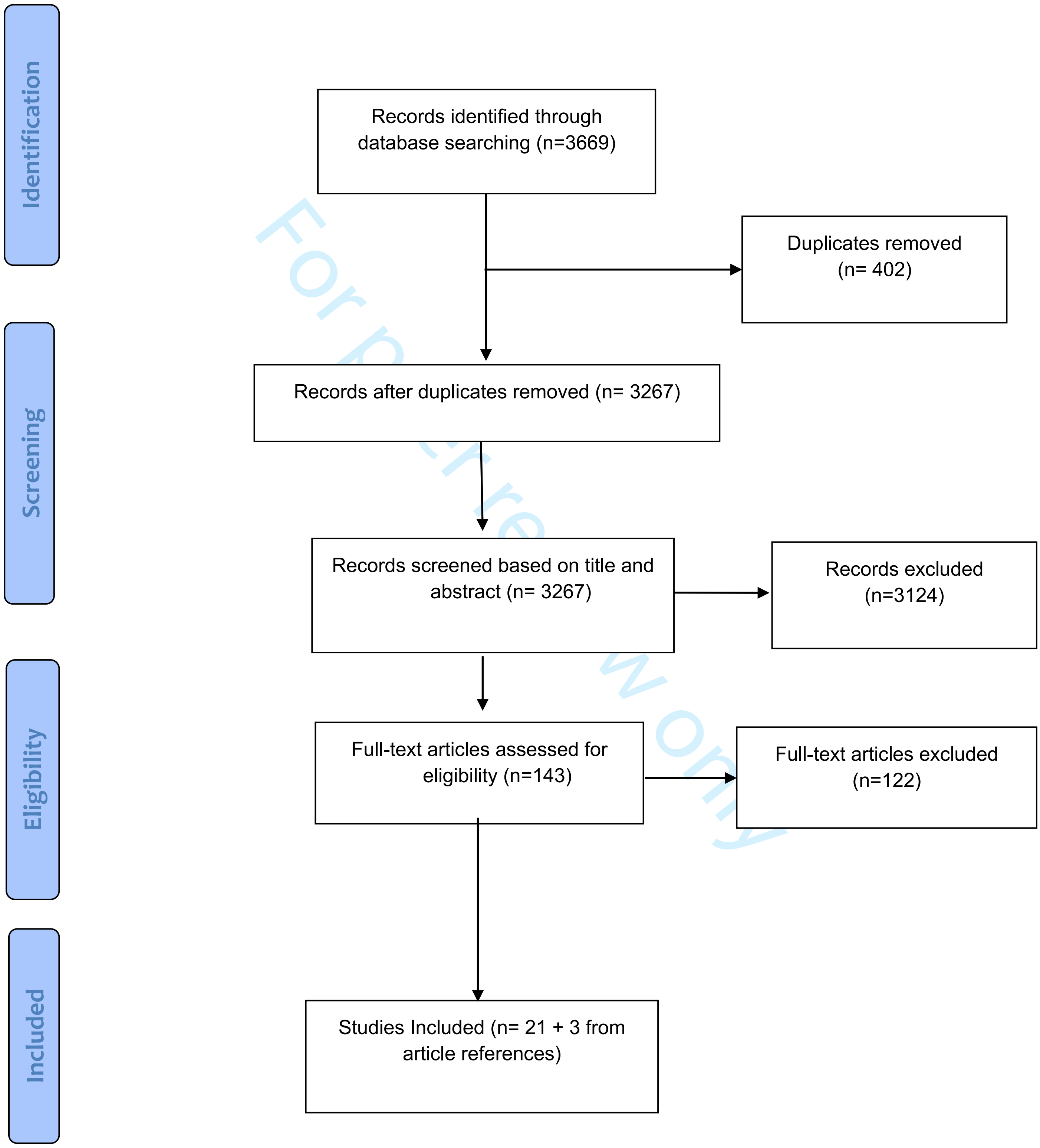

For peer review only

Supplementary Materials

Supplementary Material 1: Full electronic search strategy by database

Database: PUBMED
Limitations:
- 2011 – Present
- Humans
- English
- Adolescent 13-18

Search Terms:
((((((((((((((((((adolescent[Title/Abstract]) OR (adolescent[MeSH Major Topic])) OR (youth[Title/Abstract]) OR (teenager[Title/Abstract])) OR ("young adult"[Title/Abstract])) OR (young adult[MeSH Major Topic])) AND ("mental health"[Title/Abstract])) OR (mental health[MeSH Major Topic])) OR ("mental illness"[Title/Abstract])) OR ("mental disorder"[Title/Abstract])) OR (mental disorders[MeSH Major Topic])) AND ("social media"[Title/Abstract])) OR (social media[MeSH Major Topic])) OR ("social network*"[Title/Abstract])) OR (social networking[MeSH Major Topic])) AND (recruit*[Title/Abstract])) OR (advert*[Title/Abstract])) OR (advertising[MeSH Major Topic]))

Database: SCOPUS
Limitations:
- 2011 – Present
- English

TITLE-ABS (adolescent OR teenager OR youth OR "young adult") AND ("mental health" OR "mental illness" OR "mental disorder") AND ("social media" OR "social network*") AND (recruit* OR advert*)

Database: COCHRANE LIBRARY
Limitations:
- 2011 – Present
- English

TITLE-ABS (adolescent OR teenager OR youth OR "young adult") AND ("mental health" OR "mental illness" OR "mental disorder") AND ("social media" OR "social network*") AND (recruit* OR advert*)

Database: Medline (through EBSCO host)
Limitations:
- 2011 – Present
- English

For peer review only

- All child – 0-18 years

(adolescent OR teenager OR youth OR "young adult") AND ("mental health" OR "mental illness" OR "mental disorder") AND ("social media" OR "social network*") AND (recruit* OR advert*)

For peer review only

Supplementary File 2: Characteristics of the 24 studies included in the scoping review.

Author, year	Study design	Study aim(s)	Participants	Recruitment methods	Social media platforms	Recruitment related findings
Amon et al., 2022	Mixed methods exploratory study	To assess the acceptability, safety, user experience, and mental health benefits of a purpose-built, counsellor facilitated social networking service (SNS).	N = 154; young people aged 13-25 (86.4% were between the ages of 13 and 18)	Participants were recruited via the Kids Helpline (KHL) website and referrals from KHL counsellors, as well as through schools and KHL social media channels.	Facebook, Instagram, and Snapchat	Not specified
Bauermeister et al., 2022	Pilot randomised controlled trial	To describe the results of a pilot randomised controlled trial of imi, a web application designed to improve mental health by supporting sexual and gender minority identity affirmation, coping self-efficacy, and coping skill practice.	N = 270; sexual and gender minority youth aged 13 to 19 living in the United States (mean age = 16.49 years)	Paid for social media advertisements	Instagram	Not specified
Brawner et al., 2019	Pilot randomised controlled trial	To estimate the effect of the targeted intervention on consistent condom use, sexual activity, the number of concurrent and sequential sexual partners, and laboratory confirmed HIV/STIs at 3, 6 and 12 month follow up assessments.	N = 108; Black youth aged 14 - 17 years (mean age = 15.8 years) living in Philadelphia, USA	Community-based mental health providers, high schools, community partners (e.g., recreation centres) and provider referrals initially. Due to issues with recruitment they later expanded to include online and social media study promotion, as well as face-to-face recruitment at public venues.	Not specified	Not specified

For peer review only

Chu & Snider, 2013	Survey	To describe the effectiveness of using Facebook as a recruitment tool for medical research	N = 88; Canadian youth aged 15 - 24 years (mean age = 16.3 years)	An advert highlighting the study was targeted to Facebook members living in Canada aged 15-24	Facebook	• The average cost per final participant was $15.35.<br>• It was possible to reach a specific population across Canada using social media.<br>• Cost was relatively inexpensive and advertising efforts required minimal personnel.<br>• Facebook's performance tracking allowed optimization of advertising parameters.<br>• The total number of clicks and impressions on weekends was much higher than on weekdays
Craig et al., 2023	Survey	To examine the rates of mental health symptoms of clinical concern and substance use, and assess which COVID-19 related stressors were predictors of these symptoms and substance use in a large Canadian sample of adolescents, with comparisons across genders.	N = 809; mean age = 15.67; adolescents aged 12-18 who lived in Canada.	Recruited through advertisements on social media platforms (Facebook, Instagram) from June 17 to June 31, 2020.	Facebook and Instagram	• Recruiting through social media made targeting adolescents from minority ethnicities and low income and/or remote families without internet or data difficult.<br>• Therefore, the results may not be generalisable to these populations and caution should be taken when interpreting the results.
Fitzsimmons-Craft et al., 2021	Cross sectional survey	To examine exposure (i.e., seeing, following, posting) to body image content emphasizing a thin ideal on various social media platforms and probable ED diagnoses, ED-related quality of life, and psychiatric comorbidities among adolescents and young adult females recruited via social media who endorsed	N = 405; young adult females aged 15 - 25 years (age split by groups 15-17 and 18-25) engaged with pro-ED social media living in the USA	Instagram, Facebook, and Twitter adverts targeting English-speaking individuals in the United States who had demonstrated an interest in and/or followed accounts that were social networking about EDs or ED-related topics. On Reddit, posts were created about the study in two pro-ED related subreddits (i.e., topic-specific communities)	Facebook, Instagram, Twitter, and Reddit	• The greatest proportion of their adolescent sample (15-17 years) were recruited through Instagram at 75%<br>• Participants felt that social media could be used for study recruitment and as a way to link people with treatment.

		viewing and/or posting pro-ED online content.				
Goldbach et al., 2023	Cross-sectional online survey	To address whether: (1) whether there are differences in behavioural health patterns (i.e., depression, anxiety, and PTSD symptoms) between urban and rural sexual minority adolescents (SMA) and (2) whether these differences are mediated by the reporting of minority stress experiences, using a comprehensive 54-item measure of minority stress designed for use with adolescents.	N = 2,558; SMA in the USA between the ages of 14 and 17; mean age 15.9	Nationwide targeted paid advertisements through varying social media platforms were used to screen, invite, and enrol SMA into the current study. Respondent driven sampling also occurred.	Not specified (refer to Schrager et al., 2022)	Not specified
Kasson et al., 2021	Feasibility study	To test the feasibility of innovative outreach methods on social media for teens with EDs and to garner feedback from this population to further adapt and tailor a mental health intervention for this population.	The Discovery Group involved N = 14 female teenagers with eating disorders aged 14-17 years; The Testing Group involved N = 30 adolescents with eating disorders aged 14-17 years.	For the Discovery Group phase, Instagram and Facebook were used, specifying keywords related to body image and eating concerns (e.g., weight, shape, thin, waist). For the Testing Group this was then expanded to include Reddit and platforms including Snapchat, TikTok, and YouTube. Advertisements included static images, images with animation, and video advertisements.	Facebook, Instagram, Reddit, TikTok and Snapchat	• Snapchat was found to be the most successful platform for recruitment and Instagram was the second<br>• Platforms such as TikTok and Instagram are more heavily image based and include other features like image filters and video editing that may promote social comparisons and thin ideal
Kelleher et al., 2018	Pilot randomised controlled trial	To determine whether a social media intervention offering resources to young people displaying references to depression appropriately targeted young people with depression and was accessed	N = 25; Tumblr users aged 15-23 (mean age = 17.5 years) who posted about depression using the search term "#depress"	Tumblr messages were sent to eligible individuals.	Tumblr	• Recruitment via Tumblr was feasible<br>• Identifying participants based on depression posts on Tumblr targeted the appropriate population for this study.

For peer review only

		by, and deemed acceptable by young people.				
Kutok et al., 2021	Feasibility study	To describe the feasibility, cost-effectiveness, and generalisability of a strategy for recruiting adolescents into research studies through social media.	N = 80; Adolescents aged 13 - 17 years (mean age = 15.33 years) from the USA who spoke English; eligibility for the larger study was cybervictimisation and smartphone ownership.	Instagram was used to advertise the study using Facebook Business Manager. Two different ad campaign strategies were used to show the different ads: Reach and Traffic. “Reach” shows an ad to as many users as possible within a target audience. “Traffic” targets users that often click on links within ad. “Story” ads are featured in Instagram stories, which are posts that typically contain a vertical 9:16 ratio video or photo that will disappear after 24 hours. “Feed” ads are featured on Instagram’s regular feed of posts and contain a square video or photo that can produce engagement by being easily liked or shared.	Instagram	• The lowest cost strategy was Traffic campaign + Feed ad placement which was $19 per participant<br>• The most popular title was "Help us learn about online drama" rather than "Tell us about your experience with online drama" and "Stand up against online drama"
Lattie et al., 2017	Feasibility study	To gather information about the feasibility and acceptability of ProjectTECH programme	N = 39; high school students aged 14 - 19 years with depression and substance abuse.	Advertisements on Instagram, through schools and other community settings.	Instagram	• Adolescents responded primarily to social media advertisements.<br>• 28 of the 40 eligible participants were referred to the study through Instagram advertisement.
McGuine et al., 2021a	Cross sectional survey	To describe the health of athletes during COVID-19-related school closures and sport cancellations; to assess whether health and wellbeing differed by sex, grade, type of sport(s) played and socioeconomic status.	N = 13002; adolescent athletes from the USA aged 13-19 years (mean age = 16.3 years)	Links to Facebook and Twitter accounts were provided to sports medicine provider colleagues across the United States and to the National Federation of State High School Associations, which passed the links to each USA state high school athletic association.	Facebook and Twitter	• The sample may be biased towards athletes from higher socioeconomic families with easy access to Internet services and social media platforms

For peer review only

McGuine et al., 2021b	Cross sectional survey	To identify changes in the health (mental health, physical activity, and quality of life) of athletes that occurred during the COVID-19 pandemic.	N = 3243 (cohort 1) and N = 5231 (cohort 2); adolescent athletes aged 13 - 19 from Wisconsin, USA	Links to Facebook and Twitter accounts were provided to medical colleagues and the Wisconsin Interscholastic Athletic Association and Wisconsin Athletic Trainers' Association who forwarded the links to high school athletes.	Facebook and Twitter	• The sample may be biased towards athletes from higher socioeconomic families with easy access to Internet services and social media platforms.
Mechler et al., 2022	Randomised clinical trial	To compare the efficacy of IPDT with an established evidence-based treatment (ICBT) for adolescent depression.	N = 272; adolescents in Sweden between the ages of 15 and 19 who had a primary diagnosis of MDD according to the DSM-5	Participants were recruited nationwide in Sweden through advertisements on social media, as well as contacts with junior and senior high schools, youth associations, social workers, and healthcare providers.	Not specified	Not specified
Mitchell et al., 2022	Survey	To examine how the COVID-19 pandemic may be differentially impacting the well-being of sexual and gender minority (SGM) youth compared with their non-SGM counterparts.	N = 990; youth and emerging adults aged 13 - 23 (age split by groups or 13-17 and 18-23) in the USA, who were English speaking	Study advertisements were placed on social media sites.	Facebook and Instagram	Not specified
Morgan et al., 2021	Survey	To investigate young people's perceived effectiveness of different treatments for mental health problems, the professionals who delivered these, and the experience of negative effects.	N = 557; young people (aged 12 - 25, mean age 18 years) recruited from English-speaking, high-income countries with a current or past mental disorder.	Potential participants were invited to access the study website via a mix of online and offline promotion. Online promotion included links from youth mental health websites. Participants were also recruited via social media and Google ads targeted to searches for help for anxiety and depression. Hard-copy advertisements were distributed to youth mental health clinics and mental health support groups in Australia.	Facebook and Twitter	Not specified

For peer review only

Salk, et al., 2020	Feasibility study	To introduce and describe the Gender Minority Youth (GMY) study and sample; to provide evidence of the feasibility of using social media recruitment, paired with a waiver of parental consent, to recruit a large and diverse online sample of US transgender and cisgender youth, including multiple subgroups of gender minority youth.	N = 3318; Cisgender and transgender adolescents aged 14-18 years old living in the USA.	Participants were recruited via advertisements on Facebook and Instagram with an advertisement budget of $1500 and a participant incentive budget of $500. Two separate advertisements were used to recruit transgender and cisgender adolescents. All ads included pictures of racially/ethnically diverse adolescents and targeted US users aged 14–18 years. The transgender ad included additional targeting to identify users associated with "interest" labels such as Gender Identity, Genderqueer, and Transgender Activism.	Facebook and Instagram	• Social media recruitment was found to be efficient and inexpensive in reaching adolescents who belong to hidden and stigmatised groups.
Santana da Rosa et al., 2019	Survey	To investigate the influence of the portrayal of suicide in a popular web series (13 Reasons Why) on mood and behaviour	N = 7004; Brazilian adolescents aged 12-18 years (mean age = 14.8)	Participants were recruited through posts on a 13 Reasons Why-themed social media group.	Facebook	Not specified
Schrager et al., 2022	Protocol for a longitudinal component added to the Goldbach (2023) study	To examine how minority stress may change throughout the course of adolescence and how stress trajectories may predict health outcomes.	N = 1,076 (a subset of the original study (35) which involved 2,558 participants); SMA in the USA between the ages of 14 and 17	Initial participants were recruited through advertising on Facebook/Instagram and YouTube. Advertisements varied slightly by platform, but all included language asking youth to "Share Your Voice" and described basic details of the research study and incentives that participants could earn. Advertising was stratified by gender, geographic region and urbanicity.	Facebook, Instagram, and YouTube	Not specified
Smith, et al., 2020	Survey	To test whether general and minority specific variables are prospectively related to non-suicidal self-injury, suicidal ideation, suicide plans, and	N = 252 sexual and gender minority adolescents aged 14-15 years	Participants were recruited from social media platforms. Study advertisements were posted in forums related to sexual and gender minority populations. The researchers also	Instagram and Tumblr	Not specified

For peer review only

		suicidal behaviours in sexual and gender minority youth.		purchased advertisements on social media platforms, using search tags related to SGM identities.		
Stephens et al., 2020	Randomised controlled trial	To share lessons learned recruiting and enrolling participants via social media into the BRAVE study - a national, multiphase project to design and evaluate a text message and video-based behavioural intervention - and tips to support campaign engagement.	N = 1030; American teenagers and young people aged 15-24 years	We R Native social media channels. Additional recruitment place through listservs associated with tribes, tribal health organisations, Indian education and human service organisations that serve AI/AN teens and young adults. Ads were placed on Facebook and Instagram and were managed using Facebook Ads Manager. Each platform had unique specifications for Ad design, target audience and dates of deployment. "Interests" were used for Ad targeting including location, age, and people who matched specific interests (e.g., National Museum of the American Indian).	Facebook and Instagram	• Ads with a more positive tone typically received greater reach and impressions than posts simply describing the study eligibility criteria.
Szlyk et al., 2019	Cross-sectional online survey	To increase knowledge of barriers to treatment for depression among social media users.	N = 165; US residents aged 15 +, however age was split (15-17 and 18+) who had posted about feeling sad or depressed on social media accounts/groups that post about depression-related topics	A range of social media platforms and online depression forums. Several recruitment methods were used, including private messaging individuals who were networking on depression-focussed groups or who were posting about depression, posting about the study on depression-focused groups, and using advertisements targeted to individuals networking about depression-related topics.	Facebook, Instagram, Twitter, Reddit, and Tumblr	• Findings suggest that social media is a promising platform to recruit individuals with depression symptoms who want mental health support.<br>• Social media has the potential as a setting to engage persons with depression in help-seeking behaviour.
Veale et al., 2018	Survey	To document the prevalence of mental health concerns among transgender youth in Canada and make comparisons with	N = 923; Transgender youth from across Canada aged 14-	Community organisations, healthcare settings, social media, and the researchers' network.	Not specified	Not specified

For peer review only

		population-based estimates; to explore differences in the prevalence of mental health problems across gender identity subgroups.	25 years (age was split as 14-18 and 19-25 years)			
Zimmerman et al., 2018	Survey	To assess adolescents who watched 13 Reasons Why by asking how it affected them regarding bullying and suicidal ideation.	N = 2,323; Brazilian adolescents aged 15 to 17	Facebook advertising was used to reach adolescents who liked pages relating to 13 Reasons Why.	Facebook	Not specified

DSM-5 = Diagnostic and Statistical Manual Version 5; ED = eating disorder; iCBT = internet-based Cognitive Behavioural Therapy; iPDT = internet-based psychodynamic therapy; MDD = major depressive disorder; PTSD = posttraumatic stress disorder; SGM = sexual and gender minority; SMA = sexual minority adolescents.; SNS = social networking service

For peer review only

Preferred Reporting Items for Systematic reviews and Meta-Analyses extension for Scoping Reviews (PRISMA-ScR) Checklist

SECTION	ITEM	PRISMA-ScR CHECKLIST ITEM	REPORTED ON PAGE #
**TITLE**			
Title	1	Identify the report as a scoping review.	1
**ABSTRACT**			
Structured summary	2	Provide a structured summary that includes (as applicable): background, objectives, eligibility criteria, sources of evidence, charting methods, results, and conclusions that relate to the review questions and objectives.	1
**INTRODUCTION**			
Rationale	3	Describe the rationale for the review in the context of what is already known. Explain why the review questions/objectives lend themselves to a scoping review approach.	2
Objectives	4	Provide an explicit statement of the questions and objectives being addressed with reference to their key elements (e.g., population or participants, concepts, and context) or other relevant key elements used to conceptualize the review questions and/or objectives.	3
**METHODS**			
Protocol and registration	5	Indicate whether a review protocol exists; state if and where it can be accessed (e.g., a Web address); and if available, provide registration information, including the registration number.	3
Eligibility criteria	6	Specify characteristics of the sources of evidence used as eligibility criteria (e.g., years considered, language, and publication status), and provide a rationale.	4
Information sources*	7	Describe all information sources in the search (e.g., databases with dates of coverage and contact with authors to identify additional sources), as well as the date the most recent search was executed.	4
Search	8	Present the full electronic search strategy for at least 1 database, including any limits used, such that it could be repeated.	Supplementary Material
Selection of sources of evidence†	9	State the process for selecting sources of evidence (i.e., screening and eligibility) included in the scoping review.	4
Data charting process‡	10	Describe the methods of charting data from the included sources of evidence (e.g., calibrated forms or forms that have been tested by the team before their use, and whether data charting was done independently or in duplicate) and any processes for obtaining and confirming data from investigators.	5
Data items	11	List and define all variables for which data were sought and any assumptions and simplifications made.	5
Critical appraisal of individual sources of evidence§	12	If done, provide a rationale for conducting a critical appraisal of included sources of evidence; describe the methods used and how this information was used in any data synthesis (if appropriate).	Click here to enter text.
Synthesis of results	13	Describe the methods of handling and summarizing the data that were charted.	5

For peer review only - http://bmjopen.bmj.com/site/about/guidelines.xhtml

SECTION	ITEM	PRISMA-ScR CHECKLIST ITEM	REPORTED ON PAGE #
**RESULTS**			
Selection of sources of evidence	14	Give numbers of sources of evidence screened, assessed for eligibility, and included in the review, with reasons for exclusions at each stage, ideally using a flow diagram.	6
Characteristics of sources of evidence	15	For each source of evidence, present characteristics for which data were charted and provide the citations.	6
Critical appraisal within sources of evidence	16	If done, present data on critical appraisal of included sources of evidence (see item 12).	Click here to enter text.
Results of individual sources of evidence	17	For each included source of evidence, present the relevant data that were charted that relate to the review questions and objectives.	7
Synthesis of results	18	Summarize and/or present the charting results as they relate to the review questions and objectives.	15
**DISCUSSION**			
Summary of evidence	19	Summarize the main results (including an overview of concepts, themes, and types of evidence available), link to the review questions and objectives, and consider the relevance to key groups.	17
Limitations	20	Discuss the limitations of the scoping review process.	18
Conclusions	21	Provide a general interpretation of the results with respect to the review questions and objectives, as well as potential implications and/or next steps.	18
**FUNDING**			
Funding	22	Describe sources of funding for the included sources of evidence, as well as sources of funding for the scoping review. Describe the role of the funders of the scoping review.	19

JBI = Joanna Briggs Institute; PRISMA-ScR = Preferred Reporting Items for Systematic reviews and Meta-Analyses extension for Scoping Reviews.

* Where *sources of evidence* (see second footnote) are compiled from, such as bibliographic databases, social media platforms, and Web sites.

† A more inclusive/heterogeneous term used to account for the different types of evidence or data sources (e.g., quantitative and/or qualitative research, expert opinion, and policy documents) that may be eligible in a scoping review as opposed to only studies. This is not to be confused with *information sources* (see first footnote).

‡ The frameworks by Arksey and O'Malley (6) and Levac and colleagues (7) and the JBI guidance (4, 5) refer to the process of data extraction in a scoping review as data charting.

§ The process of systematically examining research evidence to assess its validity, results, and relevance before using it to inform a decision. This term is used for items 12 and 19 instead of "risk of bias" (which is more applicable to systematic reviews of interventions) to include and acknowledge the various sources of evidence that may be used in a scoping review (e.g., quantitative and/or qualitative research, expert opinion, and policy document).

From: Tricco AC, Lillie E, Zarin W, O'Brien KK, Colquhoun H, Levac D, et al. PRISMA Extension for Scoping Reviews (PRISMAScR): Checklist and Explanation. Ann Intern Med. 2018;169:467–473. doi: 10.7326/M18-0850.

For peer review only - http://bmjopen.bmj.com/site/about/guidelines.xhtml
